# Correction: Impact of taxes and warning labels on red meat purchases among US consumers: A randomized controlled trial

**DOI:** 10.1371/journal.pmed.1004357

**Published:** 2024-02-21

**Authors:** The PLOS Medicine Staff

## Notice of Republication

This article was republished on January 31, 2024, to replace incorrect Figure and Table files and correct minor errors in reported numerical values. The publisher apologizes for the errors. Please download this article again to view the correct version.
