# Supplementary material for: Impact of taxes and warning labels on red meat purchases among US consumers: A randomized controlled trial
Source: PLoS Med. 2023 Sep 18;20(9):e1004284. doi: 10.1371/journal.pmed.1004284 (PMC10545115; doi:10.1371/journal.pmed.1004284)
Supplement: S1 Appendix — (DOCX) [file pmed.1004284.s002.docx]

# S1 Appendix. Experimental setting.

Product data were scraped from this online food retailer in August 2020. Data on each product included: name, image, price, department, aisle, shelf, shelf rank, serving size, servings per container, ingredients list, nutritional information, symbol (i.e., barcode), and universal product code. Research assistants reviewed products’ nutrition information and corrected erroneous or missing information by extracting data from the grocery store’s website or the product manufacturer’s website. A team of registered dietitians compiled a list of keywords for identifying red meat products. Research assistants reviewed each products’ ingredient list and tagged each as red meat-containing (1) or not red meat-containing (0) based off of the presence of one or more keywords in the ingredients list.
