# Supplementary material for: Impact of taxes and warning labels on red meat purchases among US consumers: A randomized controlled trial
Source: PLoS Med. 2023 Sep 18;20(9):e1004284. doi: 10.1371/journal.pmed.1004284 (PMC10545115; doi:10.1371/journal.pmed.1004284)
Supplement: S2 Appendix — (DOCX) [file pmed.1004284.s003.docx]

# S2 Appendix. Moderator operationalization.

The moderators were operationalized as follows: red meat consumption in the past 30 days (once a week, two to three times a week, four to six times a week, and once a day or more), interest in health (low, moderate-low, moderate-high, high), interest in sustainability (low, moderate-low, moderate-high, high), household income in the last 12 months (less than $35 000, between $35 000 and $74 999, and more than $74 999), education (high school diploma or less, associate or technical degree, four-year college degree, and graduate degree), age (18-39y, 40-59y, 60y or over), race/ethnicity (Hispanic, any racial identity; non-Hispanic (NH) white; NH Black or African American; NH Asian or Pacific Islander; NH American Indian or Alaska Native, or other racial identity, including multiracial), political orientation (liberal, moderate, conservative), and gender (man and woman).

The interest in health and interest in sustainability scales were initially categorized based on the original 5-point agreement Likert scale (strongly disagree, somewhat agree, neither agree nor disagree, somewhat agree, and strongly agree). Because very few participants had the lowest possible interest in health, we combined ‘strongly disagree’ and ‘somewhat disagree’ into one group. Similarly, because very few participants had the highest possible interest in sustainability, we combined ‘somewhat agree’ and ‘strongly agree’ into one group. Differences by gender other than man and woman could not be examined due to small numbers of participants.
