# Supplementary material for: Impact of taxes and warning labels on red meat purchases among US consumers: A randomized controlled trial
Source: PLoS Med. 2023 Sep 18;20(9):e1004284. doi: 10.1371/journal.pmed.1004284 (PMC10545115; doi:10.1371/journal.pmed.1004284)
Supplement: S3 Appendix — (DOCX) [file pmed.1004284.s004.docx]

# S3 Appendix. Sensitivity analyses results.

Dropping out (defined as having started but not completed the shopping task) was statistically significantly associated with age, the amount of household grocery shopping done by the participant (thereafter, ‘grocery shopping’), and the amount of grocery shopping done by the participant online (thereafter, ‘online shopping’), but not statistically significantly associated with condition, gender, or red meat consumption in the past 30 days (thereafter, ‘red meat consumption’) (**S9 Table**). Since participants could answer the questionnaire only if they had completed the shopping task, it was not possible to examine whether any of the sociodemographic characteristics collected in the questionnaire (e.g., education level, household income) was associated with dropping out.

Linear regressions of the primary outcomes on condition, age, grocery shopping, and online shopping further revealed that online shopping was associated with the primary outcomes (p < 0.001), while age and grocery shopping were not (p > 0.05). That online shopping predicts both sample selection and the primary outcomes raises the possibility of bias in the parameter estimates of the Poisson and fractional probit regressions in the main analyses.

We investigated this issue in two ways. First, we estimated Heckman selection models with condition in the outcome equation and age, grocery shopping, and online shopping in the selection equation, and assessed the significance of the selection equation by a Wald test. Second, we estimated linear regressions, weighting the final sample with inverse probability weights (IPWs), i.e., the inverse of the probability of having completed the shopping task, which we estimated from a logit model with age, grocery shopping, and online shopping as predictors (after eliminating condition, gender, and red meat consumption in a stepwise fashion, starting with the least significant). Summary statistics for the inverse probability weights in the final sample are shown in **S10 Table**. The results are shown in **S11 Table** alongside the results from the sensitivity analyses described in the text.
