# Supplementary material for: Impact of taxes and warning labels on red meat purchases among US consumers: A randomized controlled trial
Source: PLoS Med. 2023 Sep 18;20(9):e1004284. doi: 10.1371/journal.pmed.1004284 (PMC10545115; doi:10.1371/journal.pmed.1004284)
Supplement: S1 Table — (DOCX) [file pmed.1004284.s005.docx]

| S1 Table. Survey measures used in the randomized trial. | | |
| --- | --- | --- |
| **Variable Name**  Variable description/source | **Item** | **Response options** |
| **Screener** | | |
| Age | How old are you? Enter your age in years. (Please enter your age using numbers, not letters) | [numeric free response; do not allow decimals] |
| Red meat screener  (Modified NHANES [1]) | **In the past 30 days, how often did you eat red meat?**    Red meat includes beef, lamb, pork, sausage, and ham. It also includes processed red meats such as bacon, hot dogs, and lunch meats, and it includes products that contain red meat (for example, canned beef soup).  It **DOES NOT** include chicken, turkey, or seafood products.  [show image of red meats] | 0=Never  1=Less than 1 time per week  2=1 time per week  3=2-3 times per week  4=4-6 times per week  5=1 time per day  6=2 times per day  7=3 or more times per day  [force response]  [if 0 or 1 is selected, redirect out of survey] |
| Household food shopping screener  (Hall et al., 2021 [2]) | How much of your household’s grocery shopping do you do? | 1=None  2=Less than half  3=About half  4=More than half  5=All  [force response]  [if 1 or 2 is selected, redirect out of survey] |
| Online shopping | In the past 30 days, how many of your groceries did you shop for online? (Online includes ordering pick-up or delivery groceries from grocery stores, supermarkets, big box stores like Walmart, Amazon, or online shopping platforms like Instacart). | 0=None  1=Less than half of my groceries  2=About half of my groceries  3=More than half of my groceries  4= All of my groceries  [force response] |
| Gender | How do you describe your gender identity? | 1=Man  2=Woman  3=Non-binary  4=Prefer to self-describe:*[free text]* |
| **Shopping task** | | |
| Shopping prompt | **You will now view an online grocery store and complete a shopping task. And then you will be complete a survey. *[put before shopping instructions]***    **The online grocery store takes about 30 seconds to load on a fast internet connection but could take up to two minutes on a slower internet connection. Thank you for your patience. *[put after shopping instructions]***  **Click “Next” to continue.** |  |
| **Post-shopping task** | | |
| Post-shopping prompt | **Thank you for completing the shopping task! You will now answer some questions.**  [page break] |  |
| **Online grocery store** | | |
| Process prompt | **The next questions are about the online grocery store.**  [page break] |  |
| Process  (Hall et al., 2021 [2]) | Overall, how difficult or easy was it to use the online grocery store? | 1=Very difficult  2=Difficult  3=Neither difficult nor easy  4=Easy  5=Very easy |
| Agreement prompt | **Say whether you agree or disagree with the following statements.** |  |
| Easily find  (Adapted from Waterlander et al. 2011 [3]) | I could easily find all of the food and beverages I was looking for in the online grocery store. | 1=Strongly disagree  2=Somewhat disagree  3=Neither agree nor disagree  4=Somewhat agree  5=Strongly agree |
| Enough  (Adapted from Waterlander et al. 2011 [3]) | There were enough food and beverage options in the online grocery store. | 1=Strongly disagree  2=Somewhat disagree  3=Neither agree nor disagree  4=Somewhat agree  5=Strongly agree |
| Felt real  (Adapted from Waterlander et al. 2011 [3]) | This online grocery store felt like a real online grocery store. | 1=Strongly disagree  2=Somewhat disagree  3=Neither agree nor disagree  4=Somewhat agree  5=Strongly agree |
| Shopping task - real store | If I completed this shopping task in a real store, I would pick similar products as I chose in this study. | 1=Strongly disagree  2=Somewhat disagree  3=Neither agree nor disagree  4=Somewhat agree  5=Strongly agree |
| Similar purchases | The foods I selected in this online grocery store were similar to my usual grocery purchases.  [page break] | 1=Strongly disagree  2=Somewhat disagree  3=Neither agree nor disagree  4=Somewhat agree  5=Strongly agree |
| Reasons not similar | You responded that your choices during this shopping trip may not have been similar to your usual food choices. Please select the reasons why your choices were not similar (Select all that apply)  [page break] | 1=I do not usually buy the items that were on the shopping list  2=The store did not have the types of products or brands I usually buy  3=I had trouble finding the products I wanted  4 = Other: [allow text entry]  [If answer to “Similar” is 1,2, or 3, ask this question] |
| **Product selection: Cognitive elaboration & Concern** | | |
|  | **Now we will ask you to think about the choices you made while you were shopping.**  [page break] |  |
| Cognitive elaboration – environment | How much did you think about the environmental harms of food products while you were shopping? | 1=Not at all  2=Very little  3=Somewhat  4=Quite a bit  5=A great deal |
| Cognitive elaboration – health | How much did you think about the health harms of food products while you were shopping? | 1=Not at all  2=Very little  3=Somewhat  4=Quite a bit  5=A great deal |
| Cognitive elaboration – price | How much did you think about the price of food products while you were shopping?  [page break] | 1=Not at all  2=Very little  3=Somewhat  4=Quite a bit  5=A great deal |
| **Red meat specific Qs** | | |
| Red meat prompt | **Now we will ask you some questions about eating red meat.**  Red meat includes beef, lamb, pork, sausage, and ham. It also includes processed red meats such as bacon, hot dogs, and lunch meats, and it includes products that contain red meat (for example, canned beef soup).  It **DOES NOT** include chicken, turkey, or seafood products.  [insert red meat image, see codebook appendix]  [page break] |  |
| Perceived healthfulness | How unhealthy or healthy would it be for you to eat red meat?  [randomize order of perceived healthfulness and intentions to limit meat consumption]  [page break] | 1=Very unhealthy  2=Somewhat unhealthy  3=Neither healthy nor unhealthy  4=Somewhat healthy  5=Very healthy |
| Intentions to limit meat consumption  (Adapted from Malek et al., 2019 [4]) | How much do you intend to reduce your red meat consumption in the next 30 days?  [randomize order of perceived healthfulness and intentions to limit meat consumption]  [page break] | 1=Not at all  2=Very little  3=Somewhat  4=Quite a bit  5=A great deal |
| Perceived risk – cancer (risk_cancer) | How much would eating red meat increase your risk of colon and rectal cancer?  [randomize order of risk_cancer & risk_envt] | 1=Not at all  2=Very little  3=Somewhat  4=Quite a bit  5=A great deal |
| Perceived risk – environment (risk_envt) | How much would eating red meat harm the environment?  [randomize order of risk_cancer & risk_envt] | 1=Not at all  2=Very little  3=Somewhat  4=Quite a bit  5=A great deal |
| Prompt | **Say how much you agree or disagree with the following statements.** |  |
| Public support – health warning labels (pol_hwl)  (Adapted from Vallance et al. 2020 [5]) | Red meat products should be labeled with warnings describing the link between red meat and diseases, such as colon cancer.  [randomize order of pol_hwl, pol_ewl, and pol_tax] | 1=Strongly disagree  2=Disagree  3=Neither agree nor disagree  4=Agree  5=Strongly agree |
| Public support – environmental warning labels (pol_ewl)  (Adapted from Vallance et al. 2020 [5]) | Red meat products should be labeled with warnings describing the link between red meat and environmental harms.  [randomize order of pol_hwl, pol_ewl, and pol_tax] | 1=Strongly disagree  2=Disagree  3=Neither agree nor disagree  4=Agree  5=Strongly agree |
| Public support – tax (pol_tax)  (Adapted from Vallance et al. 2020 [5]) | Red meat products should receive an extra tax to reflect the health and environmental harms of red meat consumption.  [randomize order of pol_hwl, pol_ewl, and pol_tax] | 1=Strongly disagree  2=Disagree  3=Neither agree nor disagree  4=Agree  5=Strongly agree |
| **Products from Lola’s** | | |
|  | **We will now ask you about some products. You may or may not have seen these products during the shopping task.**  [participants will see 3 products (burger patties, pepperoni pizza, ham lunch meat), randomize their order of appearance, one page per product. The participants will see the products according to their study condition in online grocery store]  [page break] |  |
| Health  (Bollard et al., 2016 [6]) | How good or bad for your health do you think this product is? | 1=Very bad  2=Somewhat bad  3=Neither bad nor good  4=Somewhat good  5=Very good |
| Environment  (Bollard et al., 2016 [6]) | How good or bad for the environment do you think this product is? | 1=Very bad  2=Somewhat bad  3=Neither bad nor good  4=Somewhat good  5=Very good |
| Cost  (Bollard et al., 2016 [6]) | How inexpensive or expensive do you think this product is?  [page break] | 1=Very inexpensive  2=Somewhat inexpensive  3=Neither inexpensive nor expensive  4=Somewhat expensive  5=Very expensive |
| **Demographics** | | |
| Prompt | **We will now ask you some more questions about yourself.**  [page break] |  |
| Ethnicity  (U.S. Census [7]) | Are you of Hispanic, Latino or Spanish origin? | 1=No, not of Hispanic, Latino, or Spanish origin  2=Yes, Mexican, Mexican American, Chicano  3=Yes, Cuban  4=Yes, another Hispanic, Latino, or Spanish origin (Enter Country of Origin): |
| Race  (U.S. Census [7]) | What is your race? (check all that apply) | 1=White  2=Black or African American  3=American Indian or Alaska Native  4=Asian  5=Pacific Islander  6=Race not listed (please specify) |
| Education  (American Community Survey [8]) | What is the highest level of education you have completed? | 1=Less than high school or U.S. high school equivalent (GED)  2=High school diploma or U.S. high school equivalent (GED)  3=Associate or technical degree  4=4-year college degree  5=Graduate degree (Master’s, PhD, or professional degree) |
| Household size | How many people live in your household, including you? | # of people [restricted to 1-20] |
| Household income [9] | Which of the following categories best describes your total household income in the last 12 months? | 1=Less than $10,000  2=$10,000 to $14,999  3=$15,000 to $24,999  4=$25,000 to $34,999  5=$35,000 to $49,999  6=$50,000 to $74,999  7=$75,000 to $99,999  8=$100,000 to $149,999  9=$150,000 to $199,999  10=$200,000 or more |
| Political affiliation | Do you consider yourself to be:  [page break] | 1=Liberal  2=Moderate  3=Conservative  [randomize order of items] |
|  | **Say how much you agree or disagree with the statements below.** |  |
| Self-perceived dietary behavior – healthy  (Hearty et al., 2006 [10]) | I make conscious efforts to try and eat a healthy diet.  [randomize order of self-perceived dietary scale] | 1=Strongly disagree  2=Somewhat disagree  3=Neither agree nor disagree  4=Somewhat agree  5=Strongly agree |
| Self-perceived dietary behavior – fat  (Hearty et al., 2006 [10]) | I try to keep the amount of fat I eat to a healthy amount. | 1=Strongly disagree  2=Somewhat disagree  3=Neither agree nor disagree  4=Somewhat agree  5=Strongly agree |
| Self-perceived dietary behavior – diet  (Hearty et al., 2006 [10]) | I do not need to change my diet as it is healthy enough.  *[page break]* | 1=Strongly disagree  2=Somewhat disagree  3=Neither agree nor disagree  4=Somewhat agree  5=Strongly agree |
|  | **Say how much you agree or disagree with the statements below.** |  |
| GREEN scale 1  (Haws et al., 2014 [11]) | It is important to me that the products I use do not harm the environment.  [randomize order of GREEN scale statements] | 1=Strongly disagree  2=Somewhat disagree  3=Neither agree nor disagree  4=Somewhat agree  5=Strongly agree |
| GREEN scale 2  (Haws et al., 2014 [11]) | I consider the potential environmental impact of my actions when making many of my decisions. | 1=Strongly disagree  2=Somewhat disagree  3=Neither agree nor disagree  4=Somewhat agree  5=Strongly agree |
| GREEN scale 3  (Haws et al., 2014 [11]) | My purchase habits are affected by my concern for the environment. | 1=Strongly disagree  2=Somewhat disagree  3=Neither agree nor disagree  4=Somewhat agree  5=Strongly agree |
| GREEN scale 4  (Haws et al., 2014 [11]) | I am concerned about wasting the resources of our planet. | 1=Strongly disagree  2=Somewhat disagree  3=Neither agree nor disagree  4=Somewhat agree  5=Strongly agree |
| GREEN scale 5  (Haws et al., 2014 [11]) | I would describe myself as environmentally responsible. | 1=Strongly disagree  2=Somewhat disagree  3=Neither agree nor disagree  4=Somewhat agree  5=Strongly agree |
| GREEN scale 6  (Haws et al., 2014 [11]) | I am willing to be inconvenienced in order to take actions that are more environmentally friendly.  *[page break]* | 1=Strongly disagree  2=Somewhat disagree  3=Neither agree nor disagree  4=Somewhat agree  5=Strongly agree |
| Combined race and ethnicity  (Modified proposed U.S. Census item [12]) | What is your race or ethnicity? (check all that apply) | 1=White  2=Hispanic, Latino, or Spanish origin  3=Black or African American  4=Asian  5=American Indian or Alaska Native  6=Middle Eastern or North African  7=Native Hawaiian or other Pacific Islander  8=Some other race or ethnicity (please specify) |
| **End** | | |
| comments | Anything you want to tell us about the study? Please leave your comments below. | [free text] |

**References**

1. Diet Behavior and Nutrition - DBQ: Centers for Disease Control and Prevention, National Center for Health Statistics; 2020 [cited 2022 December 7]. Available from: <https://wwwn.cdc.gov/nchs/data/nhanes/2019-2020/questionnaires/DBQ_K.pdf>.

2. Hall MG, Higgins ICA, Grummon AH, Lazard AJ, Prestemon CE, Sheldon JM, et al. Using a Naturalistic Store Laboratory for Clinical Trials of Point-of-Sale Nutrition Policies and Interventions: A Feasibility and Validation Study. Int J Environ Res Public Health. 2021;18(16). Epub 2021/08/28. doi: 10.3390/ijerph18168764. PubMed PMID: 34444509; PubMed Central PMCID: PMCPMC8394834.

3. Waterlander WE, Scarpa M, Lentz D, Steenhuis IH. The virtual supermarket: an innovative research tool to study consumer food purchasing behaviour. BMC Public Health. 2011;11:589. Epub 2011/07/27. doi: 10.1186/1471-2458-11-589. PubMed PMID: 21787391; PubMed Central PMCID: PMCPMC3160378.

4. Malek L, Umberger WJ, Goddard E. Committed vs. uncommitted meat eaters: Understanding willingness to change protein consumption. Appetite. 2019;138:115-26. Epub 2019/03/29. doi: 10.1016/j.appet.2019.03.024. PubMed PMID: 30917940.

5. Vallance K, Stockwell T, Zhao J, Shokar S, Schoueri-Mychasiw N, Hammond D, et al. Baseline Assessment of Alcohol-Related Knowledge of and Support for Alcohol Warning Labels Among Alcohol Consumers in Northern Canada and Associations With Key Sociodemographic Characteristics. J Stud Alcohol Drugs. 2020;81(2):238-48. Epub 2020/05/03. doi: 10.15288/jsad.2020.81.238. PubMed PMID: 32359055; PubMed Central PMCID: PMCPMC7201212.

6. Bollard T, Maubach N, Walker N, Ni Mhurchu C. Effects of plain packaging, warning labels, and taxes on young people's predicted sugar-sweetened beverage preferences: an experimental study. Int J Behav Nutr Phys Act. 2016;13(1):95. Epub 2016/09/02. doi: 10.1186/s12966-016-0421-7. PubMed PMID: 27580589; PubMed Central PMCID: PMCPMC5007687.

7. United States Census 2020: U.S. Department of Commerce, Economics and Statistics Administration, U.S. Census Bureau; 2020 [cited 2022 December 7]. Available from: <https://www2.census.gov/programs-surveys/decennial/2020/technical-documentation/questionnaires-and-instructions/questionnaires/2020-informational-questionnaire.pdf>.

8. Why we ask questions about educational attainment, undergraduate field of degree: American Community Survey, U.S. Census Bureau; 2020 [cited 2022 December 7]. Available from: <https://www.census.gov/acs/www/about/why-we-ask-each-question/education/>.

9. The Population Assessment of Tobacco and Health (PATH) Study: National Institutes of Health, National Institute on Drug Abuse, U.S. Food & Drug Administration; 2014 [cited 2022 December 7]. Available from: <https://pathstudyinfo.nih.gov/landing>.

10. Hearty A, McCarthy S, Kearney J, Gibney M. Relationship between attitudes towards healthy eating and dietary behaviour, lifestyle and demographic factors in a representative sample of Irish adults. Appetite. 2007;48(1):1-11.

11. Haws KL, Winterich KP, Naylor RW. Seeing the world through GREEN-tinted glasses: Green consumption values and responses to environmentally friendly products. Journal of Consumer Psychology. 2014;24(3):336-54.

12. Marks R, Jones N. Collecting and Tabulating Ethnicity and Race Responses in the 2020 Census: Population Division, U.S. Census Bureau; 2020 [cited 2022 December 7]. Available from: <https://www2.census.gov/about/training-workshops/2020/2020-02-19-pop-presentation.pdf>.
