# Supplementary material for: Impact of taxes and warning labels on red meat purchases among US consumers: A randomized controlled trial
Source: PLoS Med. 2023 Sep 18;20(9):e1004284. doi: 10.1371/journal.pmed.1004284 (PMC10545115; doi:10.1371/journal.pmed.1004284)
Supplement: S2 Table — aSources: Decennial Census 2020 for age, sex, race, and ethnicity and American Community Survey 2021 for household income in the past 12 months. bParticipants who completed the shopping task (n = 3,518). cSample: Sex refers to gender (sex information not collected and population statistics on gender not found). dAlone or in combination with other races. eOur questionnaire included being of Spanish origin together with Hispanic or Latino ethnicity. (DOCX) [file pmed.1004284.s006.docx]

| S2 Table. Comparison of sample statistics with population statistics. |  |  |
| --- | --- | --- |
|  | **Population ^a^**  **%** | **Sample ^b^**  **%** |
| **Age, population aged 18 years or over** |  |  |
| 18-29 | 20.7 | 17.1 |
| 30-39 | 17.2 | 17.7 |
| 40-49 | 15.7 | 15.1 |
| 50-59 | 16.6 | 17.3 |
| 60-69 | 15.3 | 19.9 |
| 70 or over | 14.5 | 12.9 |
| Total | 100.0 | 100.0 |
| **Sex, population aged 18 years or over ^c^** |  |  |
| Female | 51.5 | 60.4 |
| Male | 48.5 | 39.1 |
| Total | 100.0 | 99.6 |
| **Race, total population ^d^** |  |  |
| White | 71.0 | 82.7 |
| Black or African American | 14.2 | 11.6 |
| American Indian or Alaskan Native | 2.9 | 2.3 |
| Asian | 7.2 | 4.4 |
| Pacific Islander | 0.5 | 0.4 |
| Other | 15.1 | 2.2 |
| Total | 110.9 | 103.5 |
| **Ethnicity ^e^** |  |  |
| Hispanic or Latino | 18.7 | 10.2 |
| Not Hispanic or Latino | 81.3 | 89.8 |
| Total | 100.0 | 100.0 |
| **Household income in the past 12 months** |  |  |
| Under $15,000 | 9.9 | 9.2 |
| $15,000 to $24,999 | 7.5 | 9.1 |
| $25,000 to $34,999 | 7.8 | 12.2 |
| $35,000 to $49,999 | 11.3 | 15.3 |
| $50,000 to $74,999 | 16.8 | 21.7 |
| $75,000 to $99,999 | 12.8 | 14.0 |
| $100,000 to $149,999 | 16.3 | 11.9 |
| $150,000 to $199,999 | 7.9 | 4.4 |
| $200,000 and over | 9.8 | 2.2 |
| Total | 100.0 | 100.0 |
| ^a^ Sources: Decennial Census 2020 for age, sex, race, and ethnicity and American Community Survey 2021 for household income in the past 12 months. | | |
| ^b^ Participants who completed the shopping task (n=3,518). | | |
| ^c^ Sample: sex refers to gender (sex information not collected and population statistics on gender not found). | | |
| ^d^ Alone or in combination with other races. | | |
| ^e^ Our questionnaire included being of Spanish origin together with Hispanic or Latino ethnicity. | | |
