# Supplementary material for: Impact of taxes and warning labels on red meat purchases among US consumers: A randomized controlled trial
Source: PLoS Med. 2023 Sep 18;20(9):e1004284. doi: 10.1371/journal.pmed.1004284 (PMC10545115; doi:10.1371/journal.pmed.1004284)
Supplement: S3 Table — aSD, standard deviation. (DOCX) [file pmed.1004284.s007.docx]

| S3 Table. Primary outcome descriptive statistics by trial condition (n=3,518). | | |  |
| --- | --- | --- | --- |
| **Study Condition** | **Percent of red meat products in shopping basket** | **Count of red meat products in shopping basket** | |
|  | **Mean (SD ^a^)** | **Mean (SD ^a^)** | |
| Control | 39.0% (18.6) | 3.5 (1.7) | |
| Warning Label | 36.1% (19.8) | 3.2 (1.8) | |
| Tax | 34.0% (18.0) | 3.1 (1.6) | |
| Warning Label + Tax | 30.8% (18.5) | 2.7 (1.6) | |
| ^a^ SD = Standard Deviation. | | | |
