# Supplementary material for: Impact of taxes and warning labels on red meat purchases among US consumers: A randomized controlled trial
Source: PLoS Med. 2023 Sep 18;20(9):e1004284. doi: 10.1371/journal.pmed.1004284 (PMC10545115; doi:10.1371/journal.pmed.1004284)
Supplement: S4 Table — aAll outcomes except total calories, saturated fat, and sodium were measured on a scale of 1 to 5, with a higher value indicating a higher amount of the construct. bSD, standard deviation. (DOCX) [file pmed.1004284.s008.docx]

| S4 Table. Secondary outcome descriptive statistics by trial condition ^a^. | | | | |
| --- | --- | --- | --- | --- |
|  | **Control** | **Warning Label** | **Tax** | **Warning Label + Tax** |
|  | **Mean (SD ^b^)** | **Mean (SD)** | **Mean (SD)** | **Mean (SD)** |
| Total calories (kcal)  (n=3,518) | 9,815.9 (3,082.4) | 10,040.5 (3,501.2) | 9,632.5 (3,129.1) | 9,504.0 (2,834.6) |
| Total saturated fat (g)  (n= 3,518) | 155.5 (91.5) | 155.8 (82.0) | 145.2 (78.0) | 142.7 (83.1) |
| Total sodium (g) (n=3,518) | 18.2 (11.0) | 18.0 (6.2) | 17.6 (8.3) | 17.9 (11.0) |
| Perceived healthfulness of eating red meat  (n=3,505) | 3.3 (1.0) | 3.0 (1.1) | 3.3 (1.0) | 3.1 (1.0) |
| Perceived risk of cancer from eating red meat  (n=3,506) | 2.8 (1.0) | 3.1 (1.1) | 2.7 (1.0) | 3.0 (1.1) |
| Perceived environmental harm of eating red meat (n=3,505) | 2.7 (1.1) | 2.8 (1.1) | 2.6 (1.0) | 2.8 (1.1) |
| Thinking about the health harms of products while shopping  (n=3,505) | 2.7 (1.3) | 2.8 (1.2) | 2.6 (1.2) | 2.8 (1.2) |
| Thinking about the environmental harm of products while shopping  (n=3,504) | 2.2 (1.2) | 2.4 (1.2) | 2.1 (1.1) | 2.4 (1.2) |
| Thinking about the price of products while shopping  (n=3,507) | 4.1 (0.9) | 4.0 (0.9) | 4.1 (0.9) | 4.1 (0.9) |
| Perceived healthfulness of specific red meat products (n=3,498) | 2.9 (0.8) | 2.7 (0.9) | 2.9 (0.8) | 2.7 (0.9) |
| Perceived health sustainability of specific red meat products (n=3,498) | 2.9 (0.7) | 2.7 (0.8) | 2.9 (0.7) | 2.8 (0.8) |
| Perceived cost of burger product (n=3,492) | 3.6 (0.9) | 3.6 (1.0) | 3.9 (0.9) | 3.9 (1.0) |
| Perceived cost of pizza product (n=3,496) | 2.8 (1.0) | 2.8 (0.9) | 3.0 (0.9) | 3.0 (0.9) |
| Perceived cost of ham product (n=3,494) | 3.2 (0.9) | 3.2 (0.9) | 3.5 (1.0) | 3.5 (1.0) |
| Intention to reduce red meat consumption in the next 30 days (n=3,506) | 2.4 (1.2) | 2.5 (1.2) | 2.3 (1.1) | 2.5 (1.2) |
| ^a^ All outcomes except total calories, saturated fat, and sodium were measured on a scale of 1 to 5, with a higher value indicating a higher amount of the construct. | | | | |
| ^b^ SD, standard deviation. | | | | |
