# Supplementary material for: Impact of taxes and warning labels on red meat purchases among US consumers: A randomized controlled trial
Source: PLoS Med. 2023 Sep 18;20(9):e1004284. doi: 10.1371/journal.pmed.1004284 (PMC10545115; doi:10.1371/journal.pmed.1004284)
Supplement: S5 Table — *Bold denotes statistically significant differences between each intervention and the control at the 5% level. aDifference is control compared to intervention. bShared superscript indicates significant difference between interventions at the 5% level. cCI, confidence interval. dP values calculated using fractional probit and Poisson regression models for percent and count of red meat products, respectively. (DOCX) [file pmed.1004284.s009.docx]

| **S5 Table. Difference in primary outcomes by trial condition (n=3,518).** | | |
| --- | --- | --- |
| Study Condition | **Percent of red meat products in shopping basket ^b^**  **Mean^d^ (95% CI ^c^)** | **Count of red meat products in shopping basket ^b^**  **Mean^d^ (95% CI ^c^)** |
| Control | 39.0 (37.8, 40.3) | 3.5 (3.4, 3.6) |
| Warning Label ^a^ | -3.0*^AB^ (-4.7, -1.2) | -0.3*^AB^ (-0.4, -0.1) |
| Tax ^a^ | -5.0*^AC^ (-6.7, -3.3) | -0.4*^AC^ (-0.6, -0.3) |
| Warning Label + Tax ^a^ | -8.2*^BC^ (-10.0, -6.5) | -0.8*^BC^ (-0.9, -0.6) |
| ^*^ Bold denotes statistically significant differences between each intervention and the control at the 5% level. | | |
| ^a^ Difference is control compared to intervention. | | |
| ^b^ Shared superscript indicates significant difference between interventions at the 5% level. | | |
| ^c^ CI = Confidence Interval. | | |
| ^d^ P-values calculated using fractional probit and Poisson regression models for percent and count of red meat products, respectively. | | |
