# Supplementary material for: Impact of taxes and warning labels on red meat purchases among US consumers: A randomized controlled trial
Source: PLoS Med. 2023 Sep 18;20(9):e1004284. doi: 10.1371/journal.pmed.1004284 (PMC10545115; doi:10.1371/journal.pmed.1004284)
Supplement: S6 Table — aDifference is control compared to intervention. bWald test of equal differences with the control across the moderator’s levels. cCI, confidence interval. dBased on a scale of self-perceived dietary behavior [28]. eBased on the GREEN Scale [29]. (DOCX) [file pmed.1004284.s010.docx]

| **S6 Table. Moderation results for impact of warning labels, tax, and combined warning label + tax interventions on count of red meat purchased by demographic characteristics.** | | | | |
| --- | --- | --- | --- | --- |
|  | **Control** | **Warning Label** | **Tax** | **Warning Label + Tax** |
|  | **Count**  **(95% CI ^c^)** | **Difference ^ab^**  **(95% CI ^c^)** | **Difference ^ab^**  **(95% CI ^c^)** | **Difference ^ab^**  **(95% CI ^c^)** |
| Red meat consumption in the last 30 days (n=3,518) |  | **p = 0.735** | **p = 0.154** | **p = 0.693** |
| 1 time/week | 3.1 (2.8, 3.3) | -0.4 (-0.7, 0.0) | -0.3 (-0.7, 0.0) | -1.0 (-1.3, -0.6) |
| 2-3 times/week | 3.5 (3.4, 3.7) | -0.3 (-0.6, -0.1) | -0.4 (-0.7, -0.2) | -0.8 (-1.0, -0.5) |
| 4-6 times/week | 3.8 (3.5, 4.0) | -0.2 (-0.6, 0.1) | -0.7 (-1.1, -0.4) | -0.7 (-1.0, -0.3) |
| ≥1 time/day | 3.6 (3.3, 3.9) | -0.1 (-0.5, 0.4) | -0.1 (-0.6, 0.3) | -0.7 (-1.1, -0.3) |
| Interest in health ^d^ (n=3,490) |  | **p = 0.162** | **p = 0.726** | **p = 0.960** |
| Low | 4.0 (3.7, 4.2) | 0.1 (-0.3, 0.5) | -0.6 (-1.0, -0.2) | -0.8 (-1.2, -0.4) |
| Moderate-low | 3.7 (3.5, 4.0) | -0.3 (-0.6, 0.0) | -0.5 (-0.8, -0.2) | -0.8 (-1.1, -0.5) |
| Moderate-high | 3.4 (3.3, 3.6) | -0.4 (-0.6, -0.2) | -0.4 (-0.6, -0.2) | -0.8 (-1.0, -0.6) |
| High | 3.0 (2.7, 3.3) | -0.1 (-0.6, 0.3) | -0.4 (-0.8, 0.0) | -0.7 (-1.1, -0.2) |
| Interest in sustainability ^e^ (n=3,489) |  | **p = 0.166** | **p = 0.455** | **p = 0.935** |
| Low | 3.8 (3.3, 4.2) | 0.3 (-0.2, 0.9) | -0.2 (-0.8, 0.4) | -0.6 (-1.2, -0.0) |
| Moderate-low | 3.8 (3.6, 4.1) | -0.3 (-0.6, 0.1) | -0.5 (-0.9, -0.2) | -0.8 (-1.1, -0.4) |
| Moderate-high | 3.6 (3.4, 3.7) | -0.4 (-0.6, -0.1) | -0.6 (-0.8, -0.3) | -0.8 (-1.0, -0.5) |
| High | 3.2 (3.0, 3.4) | -0.3 (-0.6, 0.0) | -0.3 (-0.6, -0.0) | -0.8 (-1.1, -0.5) |
| Household income in the last 12 months (n=3,487) |  | **p = 0.405** | **p = 0.413** | **p = 0.496** |
| Low ($0 to < $35,000) | 3.5 (3.3, 3.7) | -0.1 (-0.4, 0.2) | -0.5 (-0.8, -0.3) | -0.9 (-1.2, -0.6) |
| Middle ($35,000 to <$74,999) | 3.6 (3.4, 3.8) | -0.4 (-0.6, -0.1) | -0.5 (-0.8, -0.2) | -0.7 (-0.9, -0.4) |
| High (≥$74,999) | 3.4 (3.2, 3.6) | -0.4 (-0.6, -0.1) | -0.3 (-0.6, -0.0) | -0.7 (-1.0, -0.5) |
| Education level (n=3,489) |  | **p = 0.611** | **p = 0.309** | **p = 0.010** |
| High school diploma or less | 3.7 (3.5, 3.9) | -0.2 (-0.5, 0.1) | -0.4 (-0.7, -0.2) | -0.8A (-1.1, -0.5) |
| Associate or technical degree | 3.6 (3.4, 3.9) | -0.3 (-0.6, 0.0) | -0.6 (-0.9, -0.3) | -0.8B (-1.2, -0.5) |
| 4-year college degree | 3.6 (3.4, 3.8) | -0.4 (-0.7, -0.1) | -0.6 (-0.8, -0.3) | -1.0C (-1.3, -0.7) |
| Graduate degree | 2.9 (2.6, 3.2) | -0.1 (-0.5, 0.3) | -0.1 (-0.5, 0.4) | -0.1ABC (-0.6, 0.3) |
| Age group (n=3,518) |  | **p = 0.618** | **p = 0.270** | **p = 0.036** |
| 18-39 | 3.5 (3.4, 3.7) | -0.4 (-0.6, -0.1) | -0.6 (-0.9, -0.4) | -1.0A (-1.2, -0.7) |
| 40-59 | 3.5 (3.3, 3.7) | -0.2 (-0.5, 0.1) | -0.4 (-0.6, -0.1) | -0.7 (-1.0, -0.4) |
| 60+ | 3.5 (3.3, 3.7) | -0.2 (-0.5, 0.1) | -0.4 (-0.6, -0.1) | -0.5A (-0.8, -0.2) |
| Race/ethnicity (n=3,490) |  | **p = 0.856** | **p = 0.981** | **p = 0.801** |
| Hispanic (any race) | 3.4 (3.1, 3.8) | -0.4 (-0.9, 0.1) | -0.5 (-1.0, -0.0) | -0.8 (-1.2, -0.3) |
| NH White | 3.5 (3.4, 3.7) | -0.2 (-0.4, -0.0) | -0.4 (-0.6, -0.2) | -0.8 (-1.0, -0.6) |
| NH Black or African American | 3.4 (2.9, 3.8) | -0.4 (-1.0, 0.2) | -0.5 (-1.1, 0.1) | -0.7 (-1.2, -0.1) |
| NH Asian or Pacific Islander | 3.1 (2.5, 3.6) | -0.6 (-1.4, 0.2) | -0.4 (-1.1, 0.3) | -0.3 (-1.1, 0.5) |
| NH Other/Multi-racial | 3.9 (3.4, 4.3) | -0.2 (-1.1, 0.7) | -0.6 (-1.3, 0.1) | -1.0 (-1.9, -0.1) |
| Political orientation (n=3,487) |  | **p = 0.628** | **p = 0.212** | **p = 0.926** |
| Liberal | 3.5 (3.3, 3.7) | -0.4 (-0.7, -0.1) | -0.6 (-0.9, -0.3) | -0.8 (-1.1, -0.5) |
| Moderate | 3.5 (3.3, 3.7) | -0.2 (-0.5, 0.1) | -0.3 (-0.5, -0.0) | -0.7 (-1.0, -0.5) |
| Conservative | 3.6 (3.4, 3.8) | -0.3 (-0.6, 0.0) | -0.5 (-0.8, -0.2) | -0.8 (-1.1, -0.5) |
| Gender (n=3,503) |  | **p = 0.190** | **p = 0.239** | **p = 0.195** |
| Woman | 3.5 (3.4, 3.6) | -0.4 (-0.5, -0.2) | -0.5 (-0.7, -0.3) | -0.8 (-1.0, -0.6) |
| Man | 3.5 (3.3, 3.7) | -0.1 (-0.4, 0.2) | -0.3 (-0.6, -0.1) | -0.6 (-0.9, -0.4) |
| ^a^ Difference is control compared to intervention.  ^b^ Wald test of equal differences with the control across the moderator’s levels.  ^c^ CI = Confidence Interval.  ^d^ Based on a scale of self-perceived dietary behavior [1].  ^e^ Based on the GREEN Scale [2]. | | | | |

**References**

1. Hearty A, McCarthy S, Kearney J, Gibney M. Relationship between attitudes towards healthy eating and dietary behaviour, lifestyle and demographic factors in a representative sample of Irish adults. Appetite. 2007;48(1):1-11.

2. Haws KL, Winterich KP, Naylor RW. Seeing the world through GREEN-tinted glasses: Green consumption values and responses to environmentally friendly products. Journal of Consumer Psychology. 2014;24(3):336-54.
