# Supplementary material for: Impact of taxes and warning labels on red meat purchases among US consumers: A randomized controlled trial
Source: PLoS Med. 2023 Sep 18;20(9):e1004284. doi: 10.1371/journal.pmed.1004284 (PMC10545115; doi:10.1371/journal.pmed.1004284)
Supplement: S7 Table — aDifference is control compared to intervention. bWald test of equal differences with the control across the moderator’s levels. cCI, confidence interval. dBased on a scale of self-perceived dietary behavior [28]. eBased on the GREEN Scale [29]. (DOCX) [file pmed.1004284.s011.docx]

| **S7 Table. Moderation results for impact of warning labels, tax, and combined warning label + tax interventions on percentage of red meat purchased by demographic characteristics.** | | | | |
| --- | --- | --- | --- | --- |
|  | **Control** | **Warning Label** | **Tax** | **Warning Label + Tax** |
|  | **Percent**  **(95% CI ^c^)** | **Difference ^ab^**  **(95% CI ^c^)** | **Difference ^ab^**  **(95% CI ^c^)** | **Difference ^ab^**  **(95% CI ^c^)** |
| Red meat consumption in the last 30 days (n=3,518) |  | **p=0.663** | **p=0.249** | **p=0.726** |
| 1 time/week | 34.1 (31.2, 37.0) | -3.5 (-7.8, 0.8) | -3.9 (-8.1, 0.3) | -10.2 (-14.2, -6.1) |
| 2-3 times/week | 39.1 (37.4, 40.8) | -4.1 (-6.6, -1.6) | -4.9 (-7.3, -2.5) | -8.6 (-11.1, -6.2) |
| 4-6 times/week | 42.0 (39.3, 44.8) | -2.1 (-5.9, 1.7) | -8.1 (-11.7, -4.5) | -7.1 (-10.9, -3.3) |
| ≥1 time/day | 40.1 (36.7, 43.6) | -0.9 (-5.9, 4.1) | -2.3 (-7.3, 2.7) | -7.5 (-12.2, -2.9) |
| Interest in health^¶^ (n=3,490) |  | **p=0.094** | **p=0.855** | **p=0.885** |
| Low | 43.6 (40.5, 46.7) | 1.1 (-3.3, 5.5) | -6.6 (-11.1, -2.1) | -8.8 (-13.3, -4.2) |
| Moderate-low | 41.4 (39.0, 43.8) | -3.3 (-6.9, 0.3) | -5.8 (-9.1, -2.6) | -8.4 (-11.8, -5.0) |
| Moderate-high | 38.8 (37.0, 40.5) | -4.9 (-7.4, -2.4) | -4.7 (-7.2, -2.2) | -9.1 (-11.5, -6.6) |
| High | 33.0 (29.7, 36.3) | -0.8 (-5.8, 4.2) | -4.4 (-9.1, 0.4) | -6.8 (-11.9, -1.6) |
| Interest in sustainability^§^ (n=3,489) |  | **p=0.051** | **p=0.441** | **p=0.762** |
| Low | 40.9 (36.3, 45.5) | 5.2 (-1.0, 11.3) | -1.5 (-7.7, 4.6) | -5.6 (-11.8, 0.7) |
| Moderate-low | 42.4 (39.7, 45.1) | -2.7 (-6.9, 1.5) | -5.9 (-9.6, -2.1) | -8.4 (-12.3, -4.5) |
| Moderate-high | 39.9 (38.1, 41.7) | -4.3 (-7.0, -1.7) | -6.3 (-8.8, -3.8) | -8.4 (-10.9, -5.8) |
| High | 35.8 (33.5, 38.1) | -3.4 (-6.7, -0.2) | -4.0 (-7.3, -0.7) | -9.4 (-12.7, -6.2) |
| Household income in the last 12 months (n=3,487) |  | **p=0.452** | **p=0.362** | **p=0.517** |
| Low ($0 to < $35,000) | 39.0 (36.7, 41.3) | -1.5 (-4.7, 1.8) | -6.2 (-9.1, -3.2) | -9.9 (-13.1, -6.8) |
| Middle ($35,000 to <$74,999) | 40.4 (38.4, 42.4) | -4.0 (-6.9, -1.1) | -5.7 (-8.5, -2.8) | -7.4 (-10.3, -4.6) |
| High (≥$74,999) | 38.0 (35.9, 40.2) | -3.9 (-7.1, -0.8) | -3.2 (-6.4, -0.1) | -8.3 (-11.4, -5.3) |
| Education level (n=3,489) |  | **p=0.497** | **p=0.172** | **p=0.006** |
| High school diploma or less | 40.8 (38.7, 42.9) | -2.4 (-5.4, 0.6) | -5.3 (-8.1, -2.5) | -8.7^A^ (-11.6, -5.7) |
| Associate or technical degree | 40.5 (38.0, 43.1) | -3.4 (-7.1, 0.2) | -6.8 (-10.3, -3.4) | -9.1^B^ (-12.7, -5.4) |
| 4-year college degree | 40.0 (37.8, 42.1) | -5.0 (-8.2, -1.8) | -6.1 (-9.2, -3.0) | -11.3^C^ (-14.4, -8.2) |
| Graduate degree | 31.9 (28.5, 35.2) | -0.8 (-5.7, 4.0) | 0.0 (-5.2, 5.2) | -1.1^ABC^ (-5.8, 3.7) |
| Age group (n=3,518) |  | **p=0.643** | **p=0.390** | **p=0.020** |
| 18-39 | 39.6 (37.6, 41.5) | -4.1 (-7.0, -1.2) | -6.7 (-9.5, -3.8) | -11.0^A^ (-13.8, -8.3) |
| 40-59 | 39.0 (36.8, 41.2) | -2.2 (-5.2, 0.9) | -4.3 (-7.2, -1.3) | -8.0 (-11.0, -4.9) |
| 60+ | 38.6 (36.3, 40.8) | -2.5 (-5.8, 0.7) | -4.1 (-7.2, -1.1) | -5.0^A^ (-8.2, -1.8) |
| Race/ethnicity (n=3,490) |  | **p=0.691** | **p=0.949** | **p=0.884** |
| Hispanic (any race) | 39.2 (34.9, 43.4) | -4.6 (-10.3, 1.1) | -6.7 (-12.2, -1.1) | -8.7 (-14.2, -3.2) |
| NH White | 39.4 (38.0, 40.9) | -2.4 (-4.5, -0.3) | -4.7 (-6.7, -2.7) | -8.5 (-10.6, -6.5) |
| NH Black or African American | 37.4 (32.9, 41.8) | -5.1 (-11.3, 1.0) | -5.2 (-11.5, 1.1) | -7.3 (-13.2, -1.4) |
| NH Asian or Pacific Islander | 34.8 (29.0, 40.7) | -8.0 (-17.1, 1.2) | -4.9 (-12.5, 2.6) | -4.5 (-13.1, 4.1) |
| NH Other/Multi-racial | 42.5 (38.3, 46.7) | -2.2 (-12.0, 7.5) | -6.9 (-14.1, 0.3) | -10.6 (-20.4, -0.9) |
| Political orientation (n=3,487) |  | **p=0.549** | **p=0.226** | **p=0.960** |
| Liberal | 38.8 (36.5, 41.0) | -4.7 (-8.0, -1.5) | -7.0 (-10.1, -3.9) | -8.8 (-11.9, -5.6) |
| Moderate | 39.1 (37.2, 41.0) | -2.4 (-5.2, 0.4) | -3.3 (-6.1, -0.6) | -8.2 (-10.9, -5.4) |
| Conservative | 39.7 (37.4, 42.0) | -2.8 (-6.0, 0.5) | -5.4 (-8.4, -2.3) | -8.3 (-11.5, -5.1) |
| Gender (n=3,503) |  | **p=0.148** | **p=0.194** | **p=0.227** |
| Woman | 39.0 (37.5, 40.5) | -4.0 (-6.2, -1.8) | -5.9 (-8.0, -3.9) | -9.1 (-11.3, -7.0) |
| Man | 39.2 (37.1, 41.3) | -1.3 (-4.3, 1.8) | -3.6 (-6.5, -0.6) | -6.9 (-9.8, -4.0) |
| ^a^ Difference is control compared to intervention.  ^b^ Wald test of equal differences with the control across the moderator’s levels.  ^c^ CI = Confidence Interval.  ^d^ Based on a scale of self-perceived dietary behavior [1].  ^e^ Based on the GREEN Scale [2]. | | | | |

**References**

1. Hearty A, McCarthy S, Kearney J, Gibney M. Relationship between attitudes towards healthy eating and dietary behaviour, lifestyle and demographic factors in a representative sample of Irish adults. Appetite. 2007;48(1):1-11.

2. Haws KL, Winterich KP, Naylor RW. Seeing the world through GREEN-tinted glasses: Green consumption values and responses to environmentally friendly products. Journal of Consumer Psychology. 2014;24(3):336-54.
