# Supplementary material for: Impact of taxes and warning labels on red meat purchases among US consumers: A randomized controlled trial
Source: PLoS Med. 2023 Sep 18;20(9):e1004284. doi: 10.1371/journal.pmed.1004284 (PMC10545115; doi:10.1371/journal.pmed.1004284)
Supplement: S8 Table — *Asterisk indicates statistically significant differences between each intervention and the control at the 5% level. aShared superscript indicates a statistically significant difference between interventions at the 5% level. bCI, confidence interval. (DOCX) [file pmed.1004284.s012.docx]

| S8 Table. Differences in policy support and opposition by trial arm (n=3,502). | | | | |
| --- | --- | --- | --- | --- |
|  | **Control** | **Warning Label** | **Tax** | **Warning Label + Tax** |
|  | **% (95% CI ^b^)** | **Percentage point (p.p) difference ^a^**  **(95% CI ^b^)** | **p.p. difference ^a^**  **(95% CI ^b^)** | **p.p. difference ^a^**  **(95% CI ^b^)** |
| **Support:**  ***Agree/strongly agree with…*** |  |  |  |  |
| A tax on red meat | 21.4 (18.7, 24.1) | 1.0^A^ (-2.8, 4.9) | -5.6*^AB^ (-9.3, -2.0) | 0.4^B^ (-3.5, 4.2) |
| A health warning label on red meat | 42.1 (38.9, 45.4) | 7.3*^A^ (2.6, 11.9) | -4.3^AB^ (-8.9, 0.3) | 6.3*^B^ (1.6, 11.0) |
| An environmental warning label on red meat | 40.2 (37.0, 43.4) | 4.7*^A^ (0.1, 9.3) | -7.1*^AB^ (-11.6, -2.6) | 4.1^B^ (-0.6, 8.7) |
| **Opposition:**  ***Disagree/strongly disagree with…*** |  |  |  |  |
| A tax on red meat | 55.7  (52.4, 59.0) | -1.0^A^  (-5.7, 3.6) | 6.9*^A^  (2.3, 11.5) | 2.3  (-2.3, 7.0) |
| A health warning label on red meat | 28.5  (25.6, 31.5) | -3.3^A^  (-7.4, 0.8) | 5.3*^AB^  (1.0, 9.6) | -1.3^B^  (-5.5, 2.9) |
| An environmental warning label on red meat | 32.2  (29.1, 35.2) | -2.9^A^  (-7.2, 1.5) | 3.2^A^  (-1.3, 7.6) | -0.8  (-5.1, 3.6) |
| ^*^ Asterisk indicates statistically significant differences between each intervention and the control at the 5% level. | | | | |
| ^a^ Shared superscript indicates a statistically significant difference between interventions at the 5% level. | | | | |
| ^b^ CI = Confidence Interval. | | | | |
