# Supplementary material for: Impact of taxes and warning labels on red meat purchases among US consumers: A randomized controlled trial
Source: PLoS Med. 2023 Sep 18;20(9):e1004284. doi: 10.1371/journal.pmed.1004284 (PMC10545115; doi:10.1371/journal.pmed.1004284)
Supplement: S9 Table — aPearson’s χ2 (chi-squared) test. bExpected counts (rounded to the nearest integer) in parentheses (only shown if p < 0.05). (DOCX) [file pmed.1004284.s013.docx]

| **S9 Table. Screener characteristics by shopping task completion status among participants who started the shopping task (n=4,106).** | | | |
| --- | --- | --- | --- |
|  | **Incomplete** | **Complete** | **p ^a^** |
| **Condition** |  |  | 0.328 |
| Control | 139 | 887 |  |
| Warning Label | 135 | 891 |  |
| Tax | 153 | 874 |  |
| Warning Label + Tax | 161 | 866 |  |
| **Age ^b^** |  |  | 0.000 |
| 18-39 | 102 (190) | 1222 (1134) |  |
| 40-59 | 127 (182) | 1141 (1086) |  |
| 60 or over | 359 (217) | 1155 (1297) |  |
| **Gender** |  |  | 0.361 |
| Woman | 334 | 2126 |  |
| Man | 252 | 1377 |  |
| Non-binary | 2 | 13 |  |
| Self-described | 0 | 2 |  |
| **Household grocery shopping done by the participant ^b^** |  |  | 0.011 |
| About half | 111 (94) | 543 (560) |  |
| More than half | 73 (94) | 580 (560) |  |
| All | 404 (401) | 2395 (2398) |  |
| **Household grocery shopping done by the participant online ^b^** |  |  | 0.000 |
| None | 375 (281) | 1587 (1681) |  |
| Less than half | 66 (116) | 745 (695) |  |
| About half | 47 (76) | 486 (457) |  |
| More than half | 30 (52) | 334 (312) |  |
| All | 70 (62) | 366 (374) |  |
| **Red meat consumption in the past 30 days** |  |  | 0.143 |
| 1 time per week | 104 | 525 |  |
| 2-3 times per week | 292 | 1696 |  |
| 4-6 times per week | 122 | 800 |  |
| 1 time per day or more | 70 | 497 |  |
| ^a^ Pearson’s χ^2^ (chi-squared) test. | | | |
| ^b^ Expected counts (rounded to the nearest integer) in parentheses (only shown if p < 0.05). | | | |
