# Supplementary material for: Impact of taxes and warning labels on red meat purchases among US consumers: A randomized controlled trial
Source: PLoS Med. 2023 Sep 18;20(9):e1004284. doi: 10.1371/journal.pmed.1004284 (PMC10545115; doi:10.1371/journal.pmed.1004284)
Supplement: S10 Table — (DOCX) [file pmed.1004284.s014.docx]

# S10 Table. Summary statistics for the inverse probability weights in the final sample (n=3,518).

| Mean | SD | Min | 1% | 5% | 10% | 25% | 50% | 75% | 90% | 95% | 99% | Max |
| --- | --- | --- | --- | --- | --- | --- | --- | --- | --- | --- | --- | --- |
| 1.17 | 0.12 | 1.04 | 1.04 | 1.06 | 1.06 | 1.07 | 1.13 | 1.22 | 1.35 | 1.42 | 1.42 | 1.50 |
