# Supplementary material for: Impact of taxes and warning labels on red meat purchases among US consumers: A randomized controlled trial
Source: PLoS Med. 2023 Sep 18;20(9):e1004284. doi: 10.1371/journal.pmed.1004284 (PMC10545115; doi:10.1371/journal.pmed.1004284)
Supplement: S11 Table — *Asterisk denotes statistically significant differences between each intervention and the control at the 5% level. aSuperscripts indicate pairwise differences between intervention conditions at the 5% level. bCI, confidence interval. cP values calculated using Poisson and fractional probit regression models for the count and percent of red meat items. dThe median completion time (shopping task and questionnaire) was 18 minutes and 46 seconds. eAll other participants (i.e., participants who purchased at least 5 of the 9 shopping list items and potentially other items not on the shopping list) were included. (DOCX) [file pmed.1004284.s015.docx]

| S11 Table. Sensitivity analyses results. | | | | |
| --- | --- | --- | --- | --- |
|  | **Control** | **Warning Label** | **Tax** | **Warning Label + Tax** |
|  | **Mean**  **(95% CI ^b^)** | **Difference ^a^**  **(95% CI ^b^)** | **Difference ^a^**  **(95% CI ^b^)** | **Difference ^a^**  **(95% CI ^b^)** |
| **Heckman selection model (n=3,518)** |  |  |  |  |
| Count of red meat items (p select. < 0.001) | 3.5 (3.3, 3.6) | -0.3*^AB^ (-0.4, -0.1) | -0.4*^AC^ (-0.6, -0.3) | -0.8*^BC^ (-0.9, -0.6) |
| Percent of red meat items (p select. < 0.001) | 38.8 (37.2, 40.4) | -3.0*^AB^ (-4.7, -1.2) | -5.0*^AC^ (-6.7, -3.3) | -8.2*^BC^ (-10.0, -6.5) |
| **Inverse probability weighted regressions (n=3,518)** |  |  |  |  |
| Count of red meat items | 3.5 (3.4, 3.6) | -0.3*^AB^ (-0.4, -0.1) | -0.4*^AC^ (-0.6, -0.3) | -0.7*^BC^ (-0.9, -0.6) |
| Percent of red meat items | 39.0 (37.7, 40.2) | -2.9*^AB^ (-4.7, -1.1) | -4.9*^AC^ (-6.6, -3.2) | -8.0*^BC^ (-9.8, -6.3) |
| **Removing participants in the lowest 2% of total expenditures ^c^ (n=3,448)** |  |  |  |  |
| Count of red meat items | 3.5 (3.4, 3.6) | -0.3*^AB^ (-0.4, -0.1) | -0.4*^AC^ (-0.6, -0.3) | -0.7*^BC^ (-0.9, -0.6) |
| Percent of red meat items | 39.2 (38.0, 40.4) | -2.9*^AB^ (-4.7, -1.1) | -4.9*^AC^ (-6.6, -3.1) | -8.0*^BC^ (-9.8, -6.3) |
| **Removing participants with completion times under half the median ^cd^ (n=3,235)** |  |  |  |  |
| Count of red meat items | 3.6 (3.5, 3.7) | -0.3*^AB^ (-0.4, -0.1) | -0.5*^AC^ (-0.6, -0.3) | -0.8*^BC^ (-0.9, -0.6) |
| Percent of red meat items | 39.8 (38.5, 41.1) | -3.1*^AB^ (-5.0, -1.3) | -5.4*^AC^ (-7.1, -3.6) | -8.2*^BC^ (-10.0, -6.4) |
| **Removing participants who purchased less than five of the nine shopping list items ^ce^ (n=3,199)** |  |  |  |  |
| Count of red meat items | 3.7 (3.6, 3.8) | -0.3*^AB^ (-0.4, -0.1) | -0.5*^AC^ (-0.6, -0.3) | -0.8*^BC^ (-0.9, -0.6) |
| Percent of red meat items | 41.3 (40.1, 42.4) | -3.0*^AB^ (-4.7, -1.3) | -5.4*^AC^ (-7.0, -3.8) | -8.5*^BC^ (-10.2, -6.8) |
| ^*^Asterisk denotes statistically significant differences between each intervention and the control at the 5% level. | | | | |
| ^a^ Superscripts indicate pairwise differences between intervention conditions at the 5% level. | | | | |
| ^b^ CI = Confidence Interval. | | | | |
| ^c^ P-values calculated using Poisson and fractional probit regression models for the count and percent of red meat items. | | | | |
| ^d^ The median completion time (shopping task and questionnaire) was 18 minutes and 46 seconds. | | | | |
| ^e^ All other participants (i.e., participants who purchased at least five of the nine shopping list items and potentially other items not on the shopping list) were included. | | | | |
