# Supplementary figures and images for: Impact of taxes and warning labels on red meat purchases among US consumers: A randomized controlled trial
Source: PLoS Med. 2023 Sep 18;20(9):e1004284. doi: 10.1371/journal.pmed.1004284 (PMC10545115; doi:10.1371/journal.pmed.1004284)

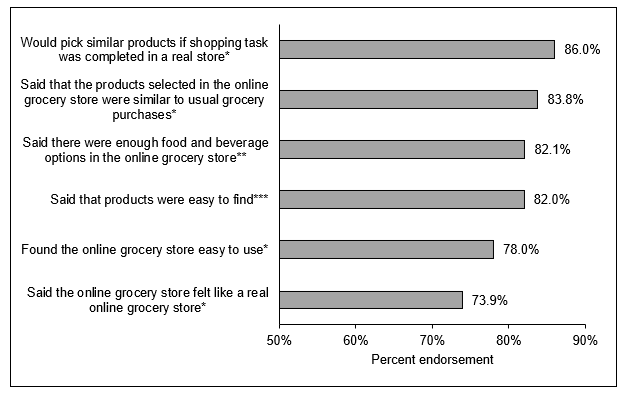

Supplement: S1 Fig — *n = 3,508; **n = 3,507; ***n = 3,509. (TIF) [file pmed.1004284.s016.tif]
