# Supplementary material for: Impact of taxes and warning labels on red meat purchases among US consumers: A randomized controlled trial
Source: PLoS Med. 2023 Sep 18;20(9):e1004284. doi: 10.1371/journal.pmed.1004284 (PMC10545115; doi:10.1371/journal.pmed.1004284)
Supplement: S1 Statistical Analysis Plan — (PDF) [file pmed.1004284.s017.pdf]

Developing and Evaluating Product Messaging

Hypotheses and Analytic Plan

NCT# NCT04716010

Version Date: 6/01/21

Hypotheses

The overall purpose of this study is to assess the impact of taxes, warnings, and a combination of taxes and warnings on US adults' decisions to purchase products that contain red meat in an online grocery store.

Primary Outcomes
The co-primary outcomes are the count of products that contain red meat and the percent of products in the shopping basket that contain red meat. We will report both count and percent of products that contain red meat.

We hypothesize that:

1. Compared to the control (no taxes and no warnings), both taxes and warnings will lead to reduced purchases of products that contain red meat.
2. The combination of taxes and warnings will lead to a larger reduction in purchases of products that contain red meat than taxes or warnings alone.

Secondary Outcomes
We hypothesize that the taxes condition, warnings condition, and taxes and warning condition will lead to:
Behavioral
A) Decrease in total saturated fat of products purchased
B) Decrease in total sodium of products purchased
C) Decrease in total calories of products purchased

Psychological
D) Decrease in perceived healthfulness of eating red meat
E) Increase in perceived risk of cancer from eating red meat
F) Increase in perceived environmental harms of eating red meat
G) Increase in thinking about the health harms of food products
H) Increase in thinking about the environmental harms of food products
I) Increase in thinking about the price of food products
J) Decrease in perceived healthfulness of specific red meat products
K) Increase in perceived environmental harm of specific red meat products
L) Increase in perceived cost of specific red meat products
M) Increase in intention to reduce red meat consumption

Other Outcomes reported by condition
We hypothesize that the taxes condition, warnings condition, and taxes and warning condition will lead to:
N) Increase in policy support for taxes on red meat products
O) Increase in policy support for health warnings on red meat products
P) Increase in policy support for environmental warnings on red meat products

Other Outcomes not reported by condition
Q) Acceptability of the online grocery store
R) Ease of use of the online grocery store

STATISTICAL CONSIDERATION

Statistical Methods

First, we will conduct a soft launch of 200 participants. With the data from the 200 participants, we will run descriptive analyses of the primary, secondary, and other outcomes, and we will confirm randomization is working properly.

If randomization is working properly and there are not apparent issues with the data collection (e.g. technical issues with the online store software), we will resume data collection until the final sample is achieved. The original 200 participants from the soft launch will be included in the final dataset.

If randomization is not working properly and/or there are apparent issues with the data collection (e.g., erroneous skip patterns), we will address all identified issues then resume data collection until the final sample is achieved. The original 200 participants from the soft launch will not be included in the final dataset.

We will descriptively report unadjusted values for primary, secondary, and other outcomes.

We will use a two-sided critical alpha of 0.05 to conduct all statistical tests. Per CONSORT guidelines, we will not test for balance in covariates. Primary analyses will be intent-to-treat, including all eligible participants with non-missing outcome data (e.g., complete-case).

Sensitivity Analyses

We will describe dropout (defined as entering the store but not completing the purchasing task) by study arm and by screener characteristic (age, red meat consumption). If differential dropout is identified, we will consider sensitivity analyses to handle missing outcome data, such as inverse probability weighting.

For all primary, secondary, and other outcomes, we will assess whether the outcomes vary by study arm with regression models, in which the referent group will be the control (compared to each of the three interventional study arms). For the primary outcomes, the regression model will be selected based on the frequency of zero purchases of products containing red meat. If the frequency of zeros is high (>10% of participants), we will consider models to deal with the high proportion of zeros (e.g., zero-inflated Poisson or zero-inflated negative binomial for a count variable; fractional regression for percentage variable). Choice of model will also depend on whether heteroscedasticity exists. Model selection exercises will be reported. For secondary and other outcomes, we will use linear regression.

For all analyses, means will be compared between each of the treatment groups and the control group, and for the primary outcome all pairwise comparisons of means will be examined. We will consider a result statistically significant at $p<0.05$.

In exploratory analyses, we will examine whether the following participant characteristics moderate the intervention effects on the primary outcomes:

a. Frequency of red meat intake
b. Interest in health
    - For interest in health, we will take the average of the 3 items if alpha>.70. The 3 items are adapted from Hearty et al. (2006). If alpha is not above .70, we will either create a scale excluding the item that makes the three-item scale drop below .70, if there is one; or we will look at the items separately.
c. Interest in sustainability
    - For interest in sustainability, we will take the average of the 6 items if alpha>.70. The six items are adapted from Haws et al. (2014). If alpha is not above .70, we will either create a scale excluding the item(s) that make(s) the six-item scale drop below .70, if there is one; or we will look at the items separately.
d. Household income level
e. Educational attainment

f. Age group
g. Race/ethnicity
h. Political orientation
i. Gender

To test whether these characteristics moderate the effect of the intervention on the count or percentage of products containing red meat selected, we will fit a series of regression models (one for each potential moderator), with trial arm, the moderator (specified as dummy variables), and their interaction as predictors. We will use a Wald chunk test to determine the joint interaction. We will quantitatively evaluate the presence of moderation by calculating the marginal effect of the intervention on the outcome at different levels of the moderating variable. If the pattern of main results is similar between intervention arms (warning label, tax, and warning plus tax), we will consider combining intervention arms for the moderation analysis.

Sample Size and Power

Sample size calculations were conducted using PASS 2019 Power Analysis and Sample Size Software (NCSS, Kaysville, Utah, USA). The effect size for the warning label was hypothesized to be the smallest effect size, and so the sample size was chosen to power this effect. With this sample size, the power associated with the tax and combined conditions will be maintained at approximately the same level or higher (better).

For a factorial design with two factors at 2 and 2 levels, assuming a Cohen's d of 0.1 (considered "small"(Cohen, 1988), and conservative relative to SSB warning label experiments which found 0.25 (Grummon & Hall, 2020)), the sample size per arm needed to achieve 80% power at an alpha level of 0.05 is n=786 per arm (n=3,144 total across the four arms).

Outliers and Exclusions

To complete the shopping task, a participant's shopping basket must have +/- 2 items of the total number of items on the shopping list. The are 9 items total on the shopping list, so the participant's basket must have 7-11 items.

We will conduct sensitivity analyses excluding individuals who are in the bottom 2 percentile of expenditures, those who complete the study unusually quickly (e.g., based on the distribution of time to completion as ascertained during a soft launch of the study), and individuals who are non-compliant with the shopping list (<50% of products selected comply with the shopping list).
