# Supplementary material for: Impact of taxes and warning labels on red meat purchases among US consumers: A randomized controlled trial
Source: PLoS Med. 2023 Sep 18;20(9):e1004284. doi: 10.1371/journal.pmed.1004284 (PMC10545115; doi:10.1371/journal.pmed.1004284)
Supplement: S1 Study Protocol — (PDF) [file pmed.1004284.s018.pdf]

Developing and Evaluating Product Messaging

Protocol
# NCT04716010
Version Date: 4/30/21

PROTOCOL TITLE: Developing and Evaluating Product Messaging

Lead Investigator:

Lindsey Smith Taillie, PhD

University of North Carolina at Chapel Hill

I confirm that I have read this protocol and understand it.

Principal Investigator Name: Lindsey Smith Taillie

Principal Investigator Signature: 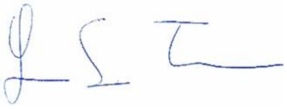

Date: 4/30/21

TABLE OF CONTENTS

Table of Contents 3
Protocol Synopsis 4
BACKGROUND AND RATIONALE 6
STUDY OBJECTIVE 6
INVESTIGATIONAL PLAN (BRIEF OVERVIEW) 7
STUDY PROCEDURES (WHAT WILL BE DONE) 9
SCREENING AND MONITORING EVALUATIONS AND MEASUREMENTS (HOW MEASUREMENTS WILL BE MADE) 10
STATISTICAL CONSIDERATION 13
SAFETY MANAGEMENT 15
DATA COLLECTION AND MANAGMENT 15
RECRUITMENT STRATEGY 15
CONSENT PROCESS 15
PLANS FOR PUBLICATION 16
REFERENCES 17

PROTOCOL SYNOPSIS

**Study Title**	Developing and Evaluating Product Messaging
**Funder**	Wellcome Trust<br>NIH National Heart, Lung, and Blood Institute
**Study Rationale**	High intake of red and processed meat is associated with both health harms, such as cancer, diabetes, and cardiovascular disease, and environmental harms, such as greenhouse gas emissions and climate change. Two potential policies to lower consumption of red and processed meat are the addition of warning labels that communicate these harms and a tax on these products. This study examines the impact of health and environmental warning labels and a red meat tax on the purchasing decisions of US meat consumers.
**Study Objective(s)**	Primary<br>• To determine the impact of a tax, warnings, and a combination of a tax and warnings on the count of products that contain red meat and the percent of products that contain red meat selected for purchase in an online grocery store.<br><br>Secondary<br>• To determine the impact of a tax, warnings, and a combination of a tax and warnings on behavioral and psychological outcomes related to red meat products in an online grocery store.
**Study Design**	Participants will electronically acknowledge their consent to participate in the study. The study platform (Gorilla Experiment Builder) will randomize each participant to one of four versions of the online grocery store (Lola's Grocery). The participants will complete a shopping task in the store based on an assigned shopping list. After completing the shopping task, the participant will complete a survey in Qualtrics. The survey will ask a series of questions about the red meat products (e.g., thinking about health/environmental harms, perceived healthfulness, perceived cost). Questions will also include standard demographic and health related variables.
**Subject Population**<br>**key criteria for Inclusion and Exclusion:**	Inclusion Criteria<br>1. 18 years old or older<br>2. Currently reside in the United States

	3. Red meat consumer (1 or more times per week)<br>4. Does at least half of the grocery shopping for their household<br>Exclusion Criteria<br>1. Participated in previous studies linked to this study
Number Of Subjects	Approximately 3,144
Study Duration	Each subject's participation will last approximately 20-30 minutes.<br>The entire study is expected to last 3 months.
Study Phases<br>Screening<br>RCT	(1) Screening: screening for eligibility and obtaining consent<br>(2) Randomization: Randomly assigning participants to one of four conditions<br>(3) Shopping task: Instruct participants to complete shopping task in an online grocery store (Lola's Grocery)<br>(4) Survey: Direct participants to a Qualtrics survey measuring perceptions and reactions to red meat warnings and a red meat tax as well as standard demographics.
Statistical And Analytic Plan	We will descriptively report unadjusted values for all primary, secondary, and other outcomes. We will examine differences between each intervention arm and the control. For primary outcomes only, we will also examine the difference between the intervention arms. In exploratory analyses, we will examine whether predetermined participant characteristics moderate the intervention effects on the primary outcomes by fitting a series of regression models, with trial arm, the moderator (specified as dummy variables), and their interaction as predictors.
DATA AND SAFETY MONITORING PLAN	The study survey will be programmed to not collect IP addresses. The research study will collect the email of every 10th participant in order to distribute a $40 gift card. However, email will be collected in a separate survey, and a randomly generated id will connect the email survey and study survey. Researchers will store the study data on a remote terminal server, and only research investigators and staff will have access to the data. Participants' emails will be shared with the panel vendor in order to distribute the $40 gift card, however study staff will not provide the panel vendor with participants' other survey responses.

BACKGROUND AND RATIONALE

Introduction

Public policy is a critical tool for improving population health. In particular, taxes and warning labels are common strategies to address unhealthy diets. For example, more than 35 countries and seven US cities now tax sugar-sweetened beverages (SSBs), and four countries have implemented nutrition warning labels on packaging, with five US states proposing similar policies.[1] These efforts have been largely successful. For example, we found that Berkeley, California, Chile, and Mexico's SSB and junk food taxes led to reductions of up to 10% in taxed product purchases, and Chile's SSB warning labels led to a 25% reduction in SSB purchases.[2-8] These SSB-focused studies show that policies like taxes and warning labels have the potential to change behavior. However, few studies have examined whether similar policies could reduce purchases of red meat in the United States, a top meat-consuming country. Given the strong associations between meat consumption and both greenhouse gas emissions and non-communicable diseases (NCDs) such as cancer, diabetes, and cardiovascular disease,[9-14] reducing meat intake in the United States is critical for mitigating climate change and preventing NCDs.

In our study, we define "red meat" as any unprocessed or processed mammalian muscle meat (e.g., beef, veal, pork, lamb, mutton, and goat).

Our proposed project aims to evaluate the effect of health and environmental warning labels and a tax on purchases of products containing red meat in an online grocery store designed to resemble a major US supermarket. Consumer behavior in virtual supermarket settings emulates shopping behavior in supermarkets,[15-18] and participants report that virtual supermarket purchases resemble regular grocery purchases.[15]

Randomized controlled trials (RCTs) are needed to test the causal impact of warnings and taxes on decisions to purchase products containing red meat. This project will address a major gap by evaluating the effect of warning labels and a tax on purchasing products containing red meat in a realistic online retail environment.

STUDY OBJECTIVE

The purpose of this study is to determine whether warning labels, a tax, or a combination of warning labels and a tax decrease purchases of products containing red meat for adult US meat consumers.

Primary Outcomes

- Percent of products purchased containing red meat
- Count of products purchased containing red meat

Secondary Outcomes

We hypothesize that the tax condition, warnings condition, and tax and warnings condition will lead to:

- Decrease in total saturated fat of products purchased
- Decrease in total sodium of products purchased
- Decrease in total calories of products purchased
- Decrease in perceived healthfulness of eating red meat
- Increase in perceived risk of cancer from eating red meat
- Increase in perceived environmental harms of eating red meat
- Increase in thinking about the health harms of food products
- Increase in thinking about the environmental harms of food products
- Increase in thinking about the price of food products
- Decrease in perceived healthfulness of specific red meat products
- Increase in perceived environmental harm of specific red meat products
- Increase in perceived cost of specific red meat products
- Increase in intention to reduce red meat consumption

Other Outcomes

We hypothesize that the tax condition, warnings condition, and tax and warnings condition will lead to:

- Increase in policy support of health and environmental warnings and taxes

We will also measure:

- Acceptability of the online grocery store
- Ease of use of the online grocery store

INVESTIGATIONAL PLAN (brief overview)

Study Design

The study design is a between-subjects randomized controlled trial. Gorilla Experiment Builder will randomly assign participants to one of four study arms in the online grocery store (Lola's Grocery). Participants will have an equal chance of being randomized to any of the trial arms.

Study Arms:

1. Control Arm: no warning labels and no tax
2. Warning Labels Arm: one black, octagonal warning label with the text: "WARNING: Eating red meat increases your risk of colon cancer and rectal cancer," and another black, octagonal warning label with the text "WARNING: Eating red meat harms the environment" on all products containing red meat (see below)

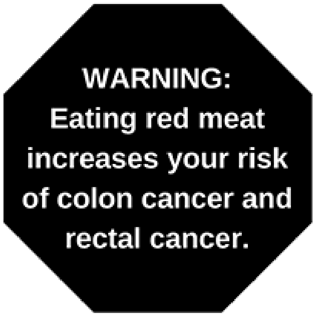

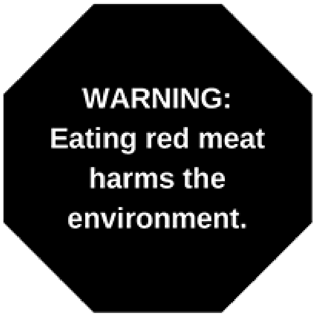

3. Tax Arm: a 30% tax on all products containing red meat
4. Combination Arm: one black, octagonal warning label with the text: “WARNING: Eating red meat increases your risk of colon cancer and rectal cancer,” one black, octagonal warning label with the text “WARNING: Eating red meat harms the environment,” and a 30% tax on all products containing red meat

In Lola’s Grocery, participants will be asked to complete a shopping task using a pre-determined shopping list. The products containing red meat in Lola’s Grocery will have a tax, warning labels, both a tax and warning labels, or neither a tax nor warning labels, depending on which arm the participant has been randomized to.

The text of the warning labels was developed through a literature review of the health and environmental harms associated with red meat consumption. The text of the warnings was narrowed down using two online experiments that tested perceived message effectiveness, level of evidence regarding the relationship between red meat consumption and harm, simplicity, and potential political feasibility. The marker word “WARNING” was included, as prior research indicates that it may increase message effectiveness. The warnings were designed to have an octagonal shape, as another study found that octagonal shapes are perceived to be more effective than rectangular labels.[19] The labels are black with white text, as this is a commonly tested color scheme for warning labels,[20] and a similar design is being used in several countries with mandated nutrient warnings, including Chile, Mexico, Peru, and Uruguay. The tax level of 30% was determined through a review of the literature for red meat tax levels that would reduce health[21-24] and environmental harms.[24,25]

After completing the shopping task, participants will complete a Qualtrics survey measuring reactions to and perceptions of red meat as well as standard demographics. Upon completion, participants will receive a pre-specified incentive from Prime Panels. Additionally, prior to entering the store, participants will be informed that 10% of participants will be randomly selected to receive the groceries they select during the shopping task and the remainder of their budget ($40) in cash. In reality, 10% of participants will be randomly selected to receive a $40 gift card. This deception is being

used to incentivize participants to select products that they would actually consume in real life.

Study Duration, Enrollment and Number of Subjects

Participation in the study will last approximately 20-30 minutes. The entire study is expected to last three months.

Study Population

Inclusion Criteria

1. 18 years old or older
2. Currently reside in the United States
3. Red meat consumer (1 or more times per week)
4. Does at least half of the grocery shopping for their household

Exclusion Criteria

1. Participation in previous studies linked to this study

STUDY PROCEDURES (what will be done)

Study Steps

Participants will be recruited through Prime Panels, a survey research firm we have worked with previously. If interested, panel members will complete a screener in the Gorilla Experiment Builder to see if they are eligible. If they are not eligible, panel members will be redirected to a termination page indicating they are not eligible to participate. If they are eligible, panel members will be redirected to a consent form. If they agree to the consent form, they will acknowledge their consent by clicking an arrow to proceed to the study.

Following online consent, participants will be randomized to one of the four arms by Gorilla and enter into Lola's Grocery. In this online grocery store, participants will complete a shopping task. They will be asked to select items using a pre-determined nine-item shopping list (see below), and they will be informed that their budget is $40. To complete the shopping task, a participant's shopping basket must have +/- 2 items of the total number of items on the shopping list (7-11 items). After completing the shopping task, participants will be redirected to a survey. In the survey, participants will answer standard behavior and reaction measurement questions, as well as standard demographic questions. Prior to the shopping task, participants will be informed that 1 in 10 participants will actually receive, via mail, the groceries they select and the remainder of their shopping budget ($40). The purpose of this is to incentivize participants to make purchases that reflect their real-life shopping habits. However, in reality, we will send 1 in 10 participants a gift card of $40 (the shopping task budget). At the end of the survey, we will inform the 1 in 10 participants that the panel vendor will be sending them a gift card

rather than groceries and ask for their email address. The panel vendor will email the gift card.

Shopping List:
1. 1 pizza
2. 1 burrito
3. Burger patties (meat or vegetarian)
4. Breakfast sausages (meat or vegetarian)
5. 1 frozen individual meal
6. 1 loaf of bread
7. 1 sandwich filling (for example, ham, turkey, or peanut butter)
8. 1 pack of tortillas
9. 1 taco filling (for example, steak, chicken, or beans)

Subject Completion/Withdrawal Procedures

A study participant is determined to have completed the study when they have finished and exited the study survey. To withdraw their data from the study, a participant would have to contact the study team or the university IRB.

Screen Failure Procedures

Prior to entering into the study, individuals will complete a survey screener. If the individual is younger than 18 years old, does not live in the United States, does not eat red meat at least one or more times per week, or does not do at least half of the grocery shopping for their household, they will be deemed ineligible. Such participants will be redirected to a screen notifying them that they are ineligible for the study.

SCREENING AND MONITORING EVALUATIONS AND MEASUREMENTS (how measurements will be made)

Measurements will include results from the shopping task as well as subjective responses by participants. The online grocery store will record how many and which items are selected for purchase, as well as how much money is spent. Secondary outcomes will include a variety of measurements, including nutrition information of the shopping basket, 5-point Likert scale questions, and yes/no questions.

Question	Response Scale
**Secondary measures**	
How unhealthy or healthy would it be for you to eat red meat?	1=Very unhealthy<br>2=Somewhat unhealthy<br>3=Neither healthy nor unhealthy

	4=Somewhat healthy<br>5=Very healthy
How much would eating red meat increase your risk of colon and rectal cancer?	1=Not at all<br>2=Very little<br>3=Somewhat<br>4=Quite a bit<br>5=A great deal
How much would eating red meat harm the environment?	1=Not at all<br>2=Very little<br>3=Somewhat<br>4=Quite a bit<br>5=A great deal
How much did you think about the environmental harms of food products while you were shopping?	1=Not at all<br>2=Very little<br>3=Somewhat<br>4=Quite a bit<br>5=A great deal
How much did you think about the health harms of food products while you were shopping?	1=Not at all<br>2=Very little<br>3=Somewhat<br>4=Quite a bit<br>5=A great deal
How much did you think about the price of food products while you were shopping?	1=Not at all<br>2=Very little<br>3=Somewhat<br>4=Quite a bit<br>5=A great deal
How good or bad for your health do you think this product is?	1=Very bad<br>2=Somewhat bad<br>3=Neither bad nor good<br>4=Somewhat good<br>5=Very good
How good or bad for the environment do you think this product is?	1=Very bad<br>2=Somewhat bad<br>3=Neither bad nor good<br>4=Somewhat good<br>5=Very good
How inexpensive or expensive do you think this product is?	1=Very inexpensive<br>2=Somewhat inexpensive<br>3=Neither inexpensive nor expensive

	4=Somewhat expensive<br>5=Very expensive
Other outcomes	
Overall, how difficult or easy was it to use the online grocery store?	1=Very difficult<br>2=Difficult<br>3=Neither difficult nor easy<br>4=Easy<br>5=Very easy
**Say whether you agree or disagree with the following statements.**	
I could easily find all of the food and beverages I was looking for in the online grocery store.	1=Strongly disagree<br>2=Somewhat disagree<br>3=Neither agree nor disagree<br>4=Somewhat agree<br>5=Strongly agree
There were enough food and beverage options in the online grocery store.	1=Strongly disagree<br>2=Somewhat disagree<br>3=Neither agree nor disagree<br>4=Somewhat agree<br>5=Strongly agree
This online grocery store felt like a real online grocery store.	1=Strongly disagree<br>2=Somewhat disagree<br>3=Neither agree nor disagree<br>4=Somewhat agree<br>5=Strongly agree
**Say how much you agree or disagree with the following statements.**	
Red meat products should be labeled with warnings describing the link between red meat and diseases, such as colon cancer.	1=Strongly disagree<br>2=Somewhat disagree<br>3=Neither agree nor disagree<br>4=Somewhat agree<br>5=Strongly agree
Red meat products should be labeled with warnings describing the link between red meat and environmental harms.	1=Strongly disagree<br>2=Somewhat disagree<br>3=Neither agree nor disagree<br>4=Somewhat agree<br>5=Strongly agree

Red meat products should receive an extra tax to reflect the health and environmental harms of red meat consumption.	1=Strongly disagree<br>2=Somewhat disagree<br>3=Neither agree nor disagree<br>4=Somewhat agree<br>5=Strongly agree

STATISTICAL CONSIDERATION

Statistical Methods

We will descriptively report unadjusted values for primary, secondary, and other outcomes.

We will use a two-sided critical alpha of 0.05 to conduct all statistical tests. Per CONSORT guidelines, we will not test for balance in covariates. Primary analyses will be intent-to-treat, including all eligible participants with non-missing outcome data (e.g., complete-case).

Sensitivity Analyses

We will describe dropout (defined as entering the store but not completing the purchasing task) by study arm and by screener characteristic (age, red meat consumption). If differential dropout is identified, we will consider sensitivity analyses to handle missing outcome data, such as inverse probability weighting.

For all primary, secondary, and other outcomes, we will assess whether the outcomes vary by study arm with regression models, in which the referent group will be the control (compared to each of the three interventional study arms). For the primary outcomes, the regression model will be selected based on the frequency of zero purchases of products containing red meat. If the frequency of zeros is high (>10% of participants), we will consider models to deal with the high proportion of zeros (e.g., zero-inflated Poisson or zero-inflated negative binomial for a count variable; fractional regression for percentage variable). Choice of model will also depend on whether heteroscedasticity exists. Model selection exercises will be reported. For secondary and other outcomes, we will use linear regression.

For all analyses, means will be compared between each of the treatment groups and the control group, and for the primary outcome all pairwise comparisons of means will be examined. We will consider a result statistically significant at $p<0.05$.

In exploratory analyses, we will examine whether the following participant characteristics moderate the intervention effects on the primary outcomes:

a. Frequency of red meat intake
b. Interest in health

    i. For interest in health, we will take the average of the 3 items if alpha>.70. The 3 items are adapted from Hearty et al. (2006).[26] If alpha is not above .70, we will either create a scale excluding the item that makes the three-item scale drop below .70, if there is one; or we will look at the items separately.

c. Interest in sustainability

    i. For interest in sustainability, we will take the average of the 6 items if alpha>.70. The six items are adapted from Haws et al. (2014).[27] If alpha is not above .70, we will either create a scale excluding the item(s) that make(s) the six-item scale drop below .70, if there is one; or, we will look at the items separately.

d. Household income level
e. Educational attainment
f. Age group
g. Race/ethnicity
h. Political orientation
i. Gender

To test whether these characteristics moderate the effect of the intervention on the count or percentage of products containing red meat selected, we will fit a series of regression models (one for each potential moderator), with trial arm, the moderator (specified as dummy variables), and their interaction as predictors. We will use a Wald chunk test to determine the joint interaction. We will quantitatively evaluate the presence of moderation by calculating the marginal effect of the intervention on the outcome at different levels of the moderating variable. If the pattern of main results is similar between intervention arms (warning label, tax, and warning plus tax), we will consider combining intervention arms for the moderation analysis.

Sample Size and Power

Sample size calculations were conducted using PASS 2019 Power Analysis and Sample Size Software (NCSS, Kaysville, Utah, USA). The effect size for the warning label was hypothesized to be the smallest effect size, and so the sample size was chosen to power this effect. With this sample size, the power associated with the tax and combined conditions will be maintained at approximately the same level or higher (better).
For a factorial design with two factors at 2 and 2 levels, assuming a Cohen's d of 0.1 (considered "small",[28] and conservative relative to SSB warning label experiments which found 0.25),[29] the sample size per arm needed to achieve 80% power at an alpha level of 0.05 is n=786 per arm (n=3,144 total across the four arms).

Outliers and Exclusions

To complete the shopping task, a participant's shopping basket must have +/- 2 items of the total number of items on the shopping list. The are 9 items total on the shopping list, so the participant's basket must have 7-11 items.

We will conduct sensitivity analyses excluding individuals who are in the bottom 2 percentile of expenditures, those who complete the study unusually quickly (e.g., based on the distribution of time to completion as ascertained during a soft launch of the study), and individuals who are non-compliant with the shopping list (<50% of products selected comply with the shopping list).

SAFETY MANAGEMENT

Provided that this study is conducted completely online, and participants do not have any interactions with study staff there is no anticipation of adverse events. However, the investigators have provided an email to contact as well as the IRB contact information, on the consent form, should a participant be concerned about any aspects of the research study.

DATA COLLECTION AND MANAGMENT

The study survey will be programmed to not collect IP addresses. The research study will collect the email of every 10th participant for gift card distribution. However, email will be collected in a separate survey, and a randomly generated id will connect the email survey and study survey. Researchers will store the study data on a remote terminal server, and only research investigators and staff will have access to the data. Participants' emails will be shared with the panel vendor in order to distribute the $40 gift card, however study staff will not provide the panel vendor with participants' other survey responses. Once gift cards are distributed, participant emails will be deleted.

RECRUITMENT STRATEGY

Participants will be Prime Panels participants who have voluntarily signed up to complete tasks remotely, including research surveys. Our study sample will include only those who self-enroll in the study based on interest, availability, and meeting the inclusion criteria. There is no specific advertisement for this study. Rather, Prime Panels identifies users for the survey based on the inclusion criteria (age 18 or older, resides in United States, consumes red meat at least once a week, and does at least 50% of household grocery shopping) and informs them of the study opportunity.

CONSENT PROCESS

To provide consent, individuals will first complete a survey screener in Qualtrics. If they are eligible for the study, they will be redirected to the study consent form. If the participant agrees to the consent form, they are informed that by continuing to the study, they are consenting to participate. The consent process is completed entirely online, and at no point in the study do participants interact with the study team.

PLANS FOR PUBLICATION

The investigators plan to publish as an open-access peer-reviewed paper. They will target public health peer-reviewed journals for manuscript submission.

REFERENCES

1. World Cancer Research Fund International. WCRF International Food Policy Framework for Healthy Diets: NOURISHING. World Cancer Reserach Fund International. http://www.wcrf.org/policy_public_affairs/nourishing_framework/index.php. Published 2014. Accessed July 20, 2014.
2. Silver LD, Ng SW, Ryan-Ibarra S, et al. Changes in prices, sales, consumer spending, and beverage consumption one year after a tax on sugar-sweetened beverages in Berkeley, California, US: A before-and-after study. *PLoS Med.* 2017;14(4):e1002283.
3. Colchero MA, Salgado JC, Unar-Munguía M, Molina M, Ng S, Rivera-Dommarco JA. Changes in Prices After an Excise Tax to Sweetened Sugar Beverages Was Implemented in Mexico: Evidence from Urban Areas. *PloS one.* 2015;10(12):e0144408.
4. Colchero MA, Rivera-Dommarco J, Popkin B, Ng SW. In Mexico, Evidence of Sustained Consumer Response Two Years after Implementing a Sugar-Sweetened Beverage Tax. *Health Affairs.* 2017;36(3):564-571.
5. Batis C, Rivera JA, Popkin B, Taillie L. First-year Evaluation of Mexico's Tax on Non-Essential Energy-Dense Foods: An Observational Study. *Plos Med.* 2016;13(7):1-14.
6. Taillie LS, Rivera J, Popkin B, Batis C. Do high vs. low purchasers respond differently to a nonessential energy-dense food tax? Two-year evaluation of Mexico's 8% nonessential food tax? *Prev Med.* 2017;105(Supplement):S37-S42.
7. Taillie LS, Colchero A, Reyes M, Popkin B, Corvalan C. Evaluation of Chilean Regulations on Front-of-Package Warning Labels and Marketing Controls on Household Purchases of Sugar-sweetened Beverages: a longitudinal study of household beverage purchases. *Under review.* 2019.
8. Caro JC, Corvalán C, Reyes M, Silva A, Popkin B, Taillie LS. Chile's 2014 sugar-sweetened beverage tax and changes in prices and purchases of sugar-sweetened beverages: An observational study in an urban environment. *PLoS medicine.* 2018;15(7):e1002597.
9. Gerber PJ, Steinfeld H, Henderson B, et al. *Tackling climate change through livestock: a global assessment of emissions and mitigation opportunities.* Food and Agriculture Organization of the United Nations (FAO); 2013.
10. Boehm R, Wilde PE, Ver Ploeg M, Costello C, Cash SB. A Comprehensive Life Cycle Assessment of Greenhouse Gas Emissions from U.S. Household Food Choices. *Food Policy.* 2018;79:67-76.
11. Clark M, Tilman D. Comparative analysis of environmental impacts of agricultural production systems, agricultural input efficiency, and food choice. *Environmental Research Letters.* 2017;12(6):064016.
12. Bouvard V, Loomis D, Guyton KZ, et al. Carcinogenicity of consumption of red and processed meat. *The Lancet Oncology.* 2015;16(16):1599-1600.
13. Chan DS, Lau R, Aune D, et al. Red and processed meat and colorectal cancer incidence: meta-analysis of prospective studies. *PloS one.* 2011;6(6):e20456.
14. Micha R, Wallace SK, Mozaffarian D. Red and processed meat consumption and risk of incident coronary heart disease, stroke, and diabetes mellitus: a systematic review and meta-analysis. *Circulation.* 2010;121(21):2271-2283.

15. Waterlander WE, Scarpa M, Lentz D, Steenhuis IH. The virtual supermarket: an innovative research tool to study consumer food purchasing behaviour. *BMC Public Health.* 2011;11:589.
16. Waterlander WE, Steenhuis IH, de Boer MR, Schuit AJ, Seidell JC. Introducing taxes, subsidies or both: the effects of various food pricing strategies in a web-based supermarket randomized trial. *Prev Med.* 2012;54(5):323-330.
17. Waterlander WE, Jiang Y, Steenhuis IH, Ni Mhurchu C. Using a 3D virtual supermarket to measure food purchase behavior: a validation study. *J Med Internet Res.* 2015;17(4):e107.
18. Ruppert B. New directions in the use of virtual reality for food shopping: marketing and education perspectives. In: SAGE Publications; 2011.
19. Grummon AH, Hall MG, Taillie LS, Brewer NT. How should sugar-sweetened beverage health warnings be designed? A randomized experiment. *Prev Med.* 2019;121:158-166.
20. Taillie LS, Hall MG, Popkin BM, Ng SW, Murukutla N. Experimental Studies of Front-of-Package Nutrient Warning Labels on Sugar-Sweetened Beverages and Ultra-Processed Foods: A Scoping Review. *Nutrients.* 2020;12(2).
21. Springmann M, Mason-D'Croz D, Robinson S, et al. Health-motivated taxes on red and processed meat: A modelling study on optimal tax levels and associated health impacts. *PLoS One.* 2018;13(11):e0204139.
22. Schönbach JK, Thiele S, Lhachimi SK. What are the potential preventive population-health effects of a tax on processed meat? A quantitative health impact assessment for Germany. (1096-0260 (Electronic)).
23. Penalvo JL, Cudhea F, Micha R, et al. The potential impact of food taxes and subsidies on cardiovascular disease and diabetes burden and disparities in the United States. *BMC Med.* 2017;15(1):208.
24. Broeks MJ, Biesbroek S, Over EAB, et al. A social cost-benefit analysis of meat taxation and a fruit and vegetables subsidy for a healthy and sustainable food consumption in the Netherlands. *BMC Public Health.* 2020;20(1):643.
25. Säll S, Gren I-M. Effects of an environmental tax on meat and dairy consumption in Sweden. *Food Policy.* 2015;55:41-53.
26. Hearty ÁP, McCarthy SN, Kearney JM, Gibney MJ. Relationship between attitudes towards healthy eating and dietary behaviour, lifestyle and demographic factors in a representative sample of Irish adults. *Appetite.* 2007;48(1):1-11.
27. Haws KL, Winterich KP, Naylor RW. Seeing the world through GREEN-tinted glasses: Green consumption values and responses to environmentally friendly products. *Journal of Consumer Psychology.* 2014;24(3):336-354.
28. Cohen J. *Statistical Power Analysis for the Behavioral Sciences.* New York, New York: New York University; 1988.
29. Grummon AH, Hall MG. Sugary drink warnings: A meta-analysis of experimental studies. *PLoS Med.* 2020;17(5):e1003120.
