# Supplementary material for: Impact of taxes and warning labels on red meat purchases among US consumers: A randomized controlled trial
Source: PLoS Med. 2023 Sep 18;20(9):e1004284. doi: 10.1371/journal.pmed.1004284 (PMC10545115; doi:10.1371/journal.pmed.1004284)
Supplement: Attachment — Submitted filename: Aim 3_Response to Reviewers_PLOS Med_Revised_FINAL_v2.docx [file pmed.1004284.s019.docx]

June 14^th^, 2023

Dear editors and reviewers,

Thank you for these thoughtful comments, which we have addressed below. We feel that addressing your points has resulted in a stronger presentation of our work. We note a few major changes including:

- We added an additional (non-pre-registered) analysis that extends the way we examine purchases of red meat. Polices targeting red meat could reduce red meat consumption by reducing the frequency of purchase (reflected in our primary outcomes, count of red meat purchases and percentage of total purchases containing red meat) as well as through reducing the total quantity of products purchased (not analyzed in our initial submission). To address the latter outcome, we added an analysis examining grams purchased from products containing red meat. We note this in the methods section (Line 188-191) and in the results section (Table 2). The corresponding reductions in grams of red meat purchases were similar to the reductions estimated using the count and percentage outcomes (i.e., a 26% relative reduction in purchases compared to a 21% or 22% relative reduction, respectively).
- We added a paragraph in the discussion about policy implications of the study.
- We added the requested author summary.

We look forward to your further review.

Best,

Lindsey Smith Taillie on behalf of co-authors

**Ms. Ref. No.:** PMEDICINE-D-23-00922R1

**Title:** “Impact of taxes and warning labels on red meat purchases in a naturalistic online grocery store: A randomized controlled trial”

| **#** | **Reviewers’ comments** | **Revision or Response** | **Line Number** |
| --- | --- | --- | --- |
|  | **Academic Editor** |  |  |
| 1 | The intro says few studies have been conducted on warning labels and taxes for meat. But there are some and I think it would be appropriate to review this literature briefly in the intro to indicate what's already known on the topic. eg. <https://bmcpublichealth.biomedcentral.com/articles/10.1186/s12889-020-08590-z> and <https://journals.plos.org/plosone/article?id=10.1371/journal.pone.0204139> Similarly, I'd like to see some of the theory around why these particular interventions - beyond simply that they've been effective in other settings - described here. Some of this is information is already in the discussion, but to my mind it is useful for the reader to have that background when considering this place of this new work as they read through it. | Thank you. We have added references to several previous studies on taxes as well as new studies on warning labels for meat in the introduction:  *Modeling studies have found that meat taxes would reduce consumption and lead to reduced mortality and healthcare costs, increased quality of life, and higher productivity [1-7].* *Similarly, one previous study evaluated graphic warning labels on meat products and found that a disgust-eliciting image under the guise of trivia reduced participants’ intention to consume meat compared to an explicit warning label [8].*  We have also added a brief discussion of the theory of these interventions in the introduction:  *Health behavior and economic theory and research indicate that these policies could lead to reductions in purchases through multiple pathways. Meat taxes reduce consumption by increasing prices and thus reducing demand for these products [9]. Warning labels reduce consumption by increasing attention, helping people understand the healthfulness or the environmental impacts associated with the product, increasing risk perceptions about harms associated with consumption, and changing behavioral intentions, leading to reductions in selecting meat products for purchase [10].* | Lines 87-91  Lines 103-108 |
| 2 | I think the sample is convenience, not purposive. If the latter, they need to be clear what purpose trying to achieve. | We agree that this sample could be considered a convenience sample and have now described it as such [11]. | Line 136 |
| 3 | Whilst I appreciate the substantial work put into this experimental naturalistic online grocery store, it remains an experimental setting. There is some mild indication in the discussion that behaviour in this may not be generalisable to real-life, but hardly any consideration of this point. It would be great to see some data on how realistic behaviour in this environment is. If that's not available, then that needs to be said. Key issues include: a minority of the population use online grocery shopping regularly so however naturalistic it is, this is not an everyday experience for them; they aren't spending their own money; they aren't responding to their 'normal' grocery shopping needs. For this reason, I find it quite hard to judge how important the findings might be. | Thanks for these useful suggestions. We have recently published a validation study [12] comparing the naturalistic online store used in this study to purchases consumers make in a real online store. We found that purchasing behavior in the naturalistic store is likely to be quite similar to purchases they would make in a real online store. For example, we found that participants’ spending on food categories in the naturalistic store were moderately-to-strongly correlated with their spending in the real store. Purchases in key food categories also mirrored national data on spending on those categories. Moreover, nearly all (95%) participants reported that their selections in the naturalistic store were similar to those made in the real online store. However, as the reviewer notes, it is unclear the extent to which other aspects of this experiment (e.g., the use of a pre-specified shopping list) may have altered purchasing behaviors and attenuated or amplified the impact of the policies.  According to our estimates [13] and more recent Gallup estimates [14], around 30 to 40% of Americans shop online for groceries. However, it is not clear the extent to which online shopping differs from shopping at a brick-and-mortar retailer and so it is unclear how generalizable our results are to consumers who do not shop online.  We have now made our discussion around these limitations clearer:  *One strength was the use of a naturalistic online grocery store designed to mimic a real-world US retailer. The use of these online stores provides a major advantage in that participants can choose from a wide assortment of real products in a setting that looks and feels like a real store. A recent validation study found that expenditures in the naturalistic online grocery store used in this study were moderately-to-strongly correlated with expenditures made in a real online grocery store, and that 95% of participants reported making similar purchases in the naturalistic online store as they did in a real online store [12]. However, in this study, it is unclear the extent to which aspects of this experiment (e.g., the use of a pre-specified shopping list, lack of financial transactions) may have altered purchasing behaviors and attenuated or amplified the impact of the policies. For example, it is possible that in the real world, where habits, preferences, and other concomitant secular trends could influence consumer response, the effects of the policies could be attenuated. Or, real-world effects could be stronger because people are spending real money.*  *In addition, our study sample differed in socio-demographic characteristics from the US population (e.g., our sample included somewhat more women and respondents who identified as non-Hispanic white), though it is unclear whether this was due to the online panel, the fact that we included only meat consumers, or both. A related limitation is that, while online food retail is growing, only about 30 to 40% of Americans shop for groceries online [13, 14]. It is not clear whether online food shopping behavior differs from in-person shopping behaviors, and thus it is unclear how generalizable our results are to consumers who do not shop online. Future research could address these limitations by testing these policies in a real-world brick-and-mortar retail setting where participants use their own shopping lists, spend their own money, and can take home products for consumption.* | Lines 412-432 |
| 4 | In terms of novelty, I'm not sure it's a major advance to show that the effect of taxes and warning labels generalise to foods they haven't explicitly commonly used previously - the paper could justify why they may or may not to help us understand this. But it is an important thing to confirm. | Thanks for this important point. We have added a paragraph in the introduction to clarify:  *Although prior research suggests that warnings and taxes reduce purchases of products like sugary drinks, tobacco, and alcohol, it is not clear whether these policies will have similar effects on red meat. In the US, meat is typically consumed as the main part of a meal [15]. Because of its more central role in the American food basket, it is possible that consumers are less likely to view red meat products as ‘discretionary’ or ‘nonessential’ as they would with sugary drinks, tobacco, or alcohol, and thus they may be less likely to change their behavior in response to taxes or labels. On the other hand, relative to sugary drinks, where the majority of Americans are aware of common health harms associated with consumption [16], a much smaller proportion are aware of the harms associated with red meat [17]. This suggests that warning labels that educate consumers about health and environmental harms may reduce consumption*. | Lines 109-117 |
|  | **Requests from the Editors** |  |  |
|  | **CLINICAL TRIALS STUDY** |  |  |
| 5 | In accordance with ICMJE requirements, PLOS Medicine requires prospective, public registration of a data sharing plan (as part of mandatory clinical trials registration) for all clinical trials that began enrollment on or after January 1, 2019. Please include a CONSORT flow chart. In the flow diagram, please indicate the number of individuals in each group analyzed in the ITT analysis. We note you currently have the flow chart as Supplementary Figure 1.; Please consider it moving to the main manuscript. | We have moved the CONSORT flow chart from the Supplementary Material to the main manuscript. The flow chart is labeled as Figure 2. | Figure 2 |
| 6 | Please include the study protocol document and analysis plan, with any amendments, as Supporting Information to be published with the manuscript if accepted. | We have included the study protocol and statistical analysis plan as Supporting Information (**S1 Statistical Analysis Plan** and **S1 Study Protocol**). | S1 Statistical Analysis Plan  S1 Study Protocol |
|  | **DATA AVAILABILITY STATEMENT** |  |  |
| 7 | Thank you for including a statement that the data is available without restriction on the Harvard Dataverse database. Please include the specific link to where the data can be found. | The dataset has been uploaded to the Harvard Dataverse. Upon publication, the following link will be made live: <https://doi.org/10.7910/DVN/HQMFUS>. | N/A |
|  | **ABSTRACT** |  |  |
| 8 | Please structure your abstract using the PLOS Medicine headings: Background, Methods and Findings, Conclusions. Please remove all other subheaders. | Done. | Lines 25-46 |
| 9 | Abstract Background: The final sentence should clearly state the study question. | We have added the following sentence to the Abstract Background:  *The main study question was, will taxes and warning labels reduce red meat purchases?* | Line 27 |
| 10 | ABSTRACT - Methods and Findings: Please provide the number in each group Please indicate the participants enrollment date(s) Please quantify the main results (with 95% CIs and p values). In the last sentence of the Abstract Methods and Findings section, please describe the main limitation(s) of the study's methodology. | We have added the numbers in each group, the participant enrollment dates, quantified the main results, and described the main limitations of the study’s methodology in the Abstract’s Methods and Findings. | Lines 28-45 |
|  | **AUTHORS SUMMARY** |  |  |
| 11 | At this stage, we ask that you include a short, non-technical Author Summary of your research to make findings accessible to a wide audience that includes both scientists and non-scientists. The Author Summary should immediately follow the Abstract in your revised manuscript. This text is subject to editorial change and should be distinct from the scientific abstract. Please see our author guidelines for more information: <https://journals.plos.org/plosmedicine/s/revising-your-manuscript#loc-author-summary>  Ideally each sub-heading should contain 2-3 single sentence, concise bullet points containing the most salient points from your study.  In the final bullet point of ‘What Do These Findings Mean?’, please include the main limitations of the study in non-technical language. | We have added this. | Lines 49-69 |
|  | **INTRODUCTION** |  |  |
| 12 | Please conclude the Introduction with a clear description of the study question (which you have done) and the approach taken to address it. | We have added this study question as follows:  *To address these gaps and inform policymaking, the main study question was, will taxes and warning labels reduce red meat purchases? To address this question, we tested the impact of taxes, warning labels, and taxes combined with warning labels on red meat purchases among US red meat consumers using a randomized controlled trial in a naturalistic online grocery store.* | Lines 118-121 |
|  | **METHODS AND RESULTS** |  |  |
| 13 | Please specify whether informed consent was written or oral (line 94) | We have updated the text to indicate that participants provided online written informed consent:  *After providing online written informed consent, participants were randomly assigned to one of four conditions in a 1:1:1:1 ratio: control (n=887), warning labels (n=891), tax (n=874), or combined (warning labels + taxes, n=866).* | Lines 146-148 |
| 14 | Please include the number of participants in each group (see design line 94-95). | We have updated the text to include the number of participants in each group.  *After providing online written informed consent, participants were randomly assigned to one of four conditions in a 1:1:1:1 ratio: control (n=887), warning labels (n=891), tax (n=874), or combined (warning labels + taxes, n=866).* | Lines 146-148 |
| 15 | Line 191: “… ANOVA to detect… “ Please define all abbreviations at first use, check and amend throughout. This includes reporting statistical information abbreviations. | We have defined the abbreviation for ANOVA and confirmed similar statistical information abbreviations are defined throughout.  *The study was originally powered for an Analysis of Variance (ANOVA) to detect a Cohen’s f=0.05 main effect of the warning labels with 80% power at the 5% level (n=786 per condition).* | Lines 231-233 |
| 16 | Please report p values as p<0.001 and where higher as p=0.002 (e.g., see line 254:  the warning label condition (p<0.01)). | We have updated the p values for the primary outcomes. | Lines 279-298 |
| 17 | Please present the primary and secondary outcomes of the study in the main paper (not in the Supporting Information files). | The primary and secondary outcomes are shown in the main manuscript. Specifically, the main outcomes on purchases are shown in Figure 3. Secondary outcomes are presented in Table 2 (nutrients purchased) and Table 3 (psychological outcomes). | Figure 3  Table 2  Table 3 |
|  | **DISCUSSION** |  |  |
| 18 | Please present and organize the Discussion as follows: a short, clear summary of the article's findings; what the study adds to existing research and where and why the results may differ from previous research; strengths and limitations of the study; implications and next steps for research, clinical practice, and/or public policy; one-paragraph conclusion. | Thanks for this comment. We confirm our discussion now follows the following organization: short summary of key findings (paragraph 1); comparison to existing literature (paragraphs 2-6); strengths and limitations (paragraph 7-8); policy implications (paragraph 9); and conclusion (paragraph 9). We note that the public health/policy implications paragraph is newly added in response to this comment, as follows:  *This study has several public health and policy implications. The results show that taxes and warning labels reduce red meat purchases among red meat consumers, suggesting that adoption and implementation of these policies could yield both health and environmental co-benefits. However, the likelihood of policy adoption is unclear. While several countries in Europe have recently considered adopting meat taxes [18-20], policy discussions on red meat in the US have been limited. A recent legal analysis found that while public policies to encourage meat reduction are legally feasible in the US, political feasibility is highly uncertain given the economic and political power of meat industry lobbies [21]. Policymakers may wish to consider the potential benefits to consumers, along with the legal and political feasibility, when weighing regulatory options for reducing intake of red and processed meat. Another barrier to policy adoption is public support: in this study, overall support for both policies was low (<50% of participants), and similar to* a *previous study [22], support was lower for taxation than labeling. However, some evidence suggests that this barrier could be overcome through different types of messaging about the policy. For example, one study in Germany found that consumers were more likely to support a meat tax if it was justified by animal welfare than by climate change mitigation [23]. Research will be necessary to understand what type of framing or justification is likely to elicit higher support for* m*eat reduction policies in the US.* | Lines 433-448 |
| 19 | Line 300-301: “To our knowledge, this was the first study to test taxes and warning labels on red meat purchases in a naturalistic online grocery store setting among US consumers”.  Please avoid assertions of primacy. | We have deleted this statement. | N/A |
|  | **ACKNOWLEDGEMENTS/DECLARATIONS** |  |  |
| 20 | Please remove all statements apart from acknowledgements, author contributions and abbreviations from the end of the main manuscript and include these only in the relevant parts of the manuscript submission form. Funding, competing interest, and data availability will be compiled as metadata. | We have removed any statements compiled as metadata. | N/A |
|  | **REFERENCES** |  |  |
| 21 | Please use the "Vancouver" style for reference formatting, and see our website for other reference guidelines <https://journals.plos.org/plosmedicine/s/submission-guidelines#loc-references> | We have updated the reference format to the Vancouver style. | Lines 469-633 |
| 22 | Please ensure that in the bibliography up to but no more than 6 author names are listed, followed by et al., in the event that more than 6 authors contribute to an individual study. Journal name abbreviations should be those found in the National Center for Biotechnology Information (NCBI) databases. | We have reviewed the bibliography to ensure that no more than 6 authors are listed for each reference. | Lines 469-633 |
|  | **FIGURES** |  |  |
| 23 | Figure 1: We note you have included images of branded (copyrighted) products. Please confirm you have appropriate permission or you may wish to remove these images. For more information, please see our guidelines: <https://journals.plos.org/plosmedicine/s/figures#loc-licenses-and-copyright> and <https://journals.plos.org/plosmedicine/s/licenses-and-copyright> | We have removed the branding from these images. | Figure 1 |
|  | **TABLES** |  |  |
| 24 | Please ensure that any and all abbreviations detailed in the tables are clearly defined in each caption/legend for the reader (e.g. see CI, for table 2,3) | We have ensured that all tables include definitions for abbreviations throughout. | Tables 1-3 |
|  | **SUPPORTING INFORMATION** |  |  |
| 25 | Figures: we note you have included images of branded products. Please remove these unless you can provide permission. For more information see <https://journals.plos.org/plosmedicine/s/figures#loc-licenses-and-copyright> and <https://journals.plos.org/plosmedicine/s/licenses-and-copyright> | We have removed the branding from these images. | S1 Table |
| 26 | Please ensure that any and all abbreviations detailed in the tables and figures are clearly defined in each caption/legend for the reader (e.g Supplemental Table 4 and 7: CI, Supplemental Table 5: SD, etc. ) | We have ensured that all abbreviations in the supplementary material tables and figures are defined in the caption/legend. | S1-S11 Table, S1 Figure |
| 27 | Please consider moving supplementary figure 1: the participant flow chart to the main manuscript. | We have moved Supplementary Figure 1 to the main manuscript (now called Figure 2). | Figure 2 |
|  | **Reviewer #1** |  |  |
| 28 | "A purposive sample of 5,533 participants was recruited in October 2021 by CloudResearch (Prime Research Solutions LLC, New York) to complete an online survey and experiment. CloudResearch targeted recruitment to create a sample that approximately represented the US population in terms of gender, race/ethnicity, income, and age." Can the authors please assess whether the final analysed cohort can be considered to be representative of the wider population, for generalisability of the study findings? | Thank you for these comments. We have added more discussion about the generalizability of study findings as follows:  *One strength was the use of a naturalistic online grocery store designed to mimic a real-world US retailer. The use of these online stores provides a major advantage in that participants can choose from a wide assortment of real products in a setting that looks and feels like a real store. A recent validation study found that expenditures in the naturalistic online grocery store used in this study were moderately-to-strongly correlated with expenditures made in a real online grocery store, and that 95% of participants reported making similar purchases in the naturalistic online store as they did in a real online store [12]. However, in this study, it is unclear the extent to which aspects of this experiment (e.g., the use of a pre-specified shopping list, lack of financial transactions) may have altered purchasing behaviors and attenuated or amplified the impact of the policies. For example, it is possible that in the real world, where habits, preferences, and other concomitant secular trends could influence consumer response, the effects of the policies could be attenuated. Or, real-world effects could be stronger because people are spending real money.*  *In addition, our study sample differed in socio-demographic characteristics from the US population (e.g., our sample included somewhat more women and respondents who identified as non-Hispanic white) , though it is unclear whether this was due to the online panel, the fact that we included only meat consumers, or both. A related limitation is that, while online food retail is growing, only about 30 to 40% of Americans shop for groceries online [13, 14]. It is not clear whether online food shopping behavior differs from shopping behaviors at traditional retailers, and thus it is unclear how generalizable our results are to consumers who do not shop online. Future research could address these limitations by testing these policies in a real-world brick-and-mortar retail setting where participants use their own shopping lists, spend their own money, and can take home products for consumption.* | Line 412-432 |
| 29 | "After providing informed consent, participants were randomly assigned to one of four conditions in a 1:1:1:1 ratio: control, warning labels, tax, or combined (warning labels + taxes)." The authors have followed a rigorous study design, helping to reduce possible sources of bias and confounding in the data and analysis. | Thank you for this comment. |  |
| 30 | "Participants completed a shopping task in the naturalistic online grocery store. Participants selected items using a pre-determined nine-item shopping list: 1 pizza, 1 burrito, burger patties (meat or vegetarian), breakfast sausages (meat or vegetarian), 1 frozen individual meal, 1 loaf of bread, 1 sandwich filling (for example, ham, turkey, or peanut butter), 1 pack of tortillas, and 1 taco filling (for example, steak, chicken, or beans). The shopping list was informed by data on the main sources of red meat consumption in the United States." and "Participants were informed that their budget was $40" The participants completed the task under the same conditions as each other, which helps to reduce potential confounding.  However, can the authors please comment on whether this may also restrict the generalisability of the study findings (i.e. the structure of the study means the research question is conditional on the shopping list and budget)? | Thanks for these important points. We have added this limitation in the discussion section, as detailed in our response to #28. | Lines 412-432 |
| 31 | "To encourage participants to select products that they would purchase in real life, participants were informed that 10% of participants would be randomly selected to receive the groceries they selected during the shopping task and the remainder of their budget in cash. In reality, 10% of participants were randomly selected to receive a $40 gift card." The approach taken by the authors seems sensible, and helps to promote more realistic shopping tendencies of the participants. | Thank you for this comment. | N/A |
| 32 | "After completing the shopping task, participants were directed to a survey to complete a post-shopping questionnaire. All participants were given an incentive paid in points redeemable by Cloud Research for completion of the survey and experiment. " Can the authors please clarify how many of the shopping participants went on to complete this survey? Can the authors please explore whether the particpants of the survey were more likely to be the participants who spent less money on the shop (e.g. were those with a tendency to save money in the shop, more prone to respond to an incentive for participation in the survey)? | That is a good question. We have added the following summary to the paper:  *Of the 3,518 participants who completed the shopping task (i.e., checked out and did not time out of the shopping task), only nine did not respond to the post-shopping-task questionnaire. Average total spending was $31.68 for those who responded to the questionnaire and $32.09 for those who did not*, *suggesting similar shopping behaviors between the two groups*. | Lines 315-318 |
| 33 | "For policy support outcomes, for simplicity of interpretation, we created a 160 binary variable, 'agree' or 'strongly agree' vs. the remaining three response options ('neither agree nor disagree', 'disagree', and 'strongly disagree')." Did the authors consider conducting a sensitivity analysis on this dichotomisation, including 'neither agree nor disagree' in the alternative categorisation for example? | This is a good suggestion. We have added a sensitivity analysis reporting the effect of each intervention both on the probability of supporting as well as the probability of opposing these policies (disagree or strongly disagree vs. other responses) (see Supplemental Table 10). We have also amended the manuscript as follows:  *The survey also assessed support for red meat taxes, health warnings, and environmental warnings. For simplicity of interpretation, we created a binary variable for support for each policy (i.e., responses of ‘agree’ or ‘strongly agree’). We also examined effects on opposition* *of these policies (responses of ‘disagree’ or ‘strongly disagree’).*  and:  *In the control condition, policy support was low for taxes (21.4% of participants in favor and 55.7% against) and higher for health and environmental warning labels on red meat (42.1% and 40.2% of participants in favor and 28.5% and 32.2% against, respectively). Exposure to the tax led to lower support for taxes and higher opposition to taxes. Exposure to the taxes also led to lower support for environmental warning labels and increased opposition to health warning labels. In contrast, exposure to the warning labels did not affect support or opposition to taxes, and increased support for both health and environmental warning labels. Exposure to the combined arm increased support for health warning labels and had no effect on support or opposition to taxes or environmental warning labels. (****S8 Table****).* | Lines 210-213  Lines 324-331 |
| 34 | "Analyses and hypotheses were pre-registered on [http://www.clinicaltrials.gov](http://www.clinicaltrials.gov/) (NCT04716010). " Can the authors please supply a copy of the study protocol and statistical analysis plan (SAP) in the next submission? | We have now included the study protocol and statistical analysis plan as Supporting Information (**S1 Statistical Analysis Plan** and **S1 Study Protocol**). | S1 Statistical Analysis Plan  S1 Study Protocol |
| 35 | "Per CONSORT guidelines, we did not test for balance in covariates across conditions" The authors have suitably provided the completed CONSORT checklist in the supplementary material. | Thanks for this comment. | N/A |
| 36 | "The study was originally powered for an ANOVA to detect a Cohen's f=0.05 main effect of the warning labels with 80% power at the 5% level (n=786 per condition). However, we chose to compare individual conditions instead, and post-hoc power calculations showed that we had a large enough sample (n=866 to 891 per condition) to detect a Cohen's d=0.13 difference between the warnings and control conditions at the 5% level with 80% power." The authors transparently communicate the basis and amendment to the sample size calculations. | Thanks. |  |
| 37 | "We used Poisson and fractional probit regression models for the count and fraction of items in the basket that contained red meat, respectively, and linear regression models for the remaining outcomes, with condition as the independent variable. We used robust standard errors in all models except linear regression models where we did not find evidence of heteroskedastic errors based on Breusch-Pagan test assuming IID errors. Differences between conditions were assessed by comparing the predicted means by condition." and "For each of these potential moderators, we fit a Poisson regression model for the count outcome and a fractional probit model for the fractional outcome with condition, the moderator, and their interaction as the independent variables. " The authors have applied technically appropriate statistical methods. | Thanks. | N/A |
| 38 | "Using the model estimates, we then estimated the difference between each intervention condition and the control condition at each level of the moderator and tested the hypothesis of equal differences with the control condition across those levels for each intervention condition, examining all pairwise comparisons (e.g., for age, 18- 39y vs. 40-59y, 18-39y vs. 60y or over, and 40-59y vs. 60y or over)." Did the authors consider correcting for multiple comparions here? | We did consider employing a multiple comparison procedure but decided not to for the following reasons. As a whole, the goal of this study was to provide early evidence about the impacts of red meat taxes and warning labels on consumer behavior to inform future research. Therefore, in general, we were more concerned about being too (rather than less) conservative. A fortiori, our moderation analyses were not meant to provide strong evidence for or against certain moderation effects, but rather to explore potential moderators for future research to study more specifically. Following Hochberg and Tamhane (1987) [24], we concluded that a ‘separate inferences approach’ (p. 11) was appropriate. | N/A |
| 39 | "As pre-registered, we carried out sensitivity analyses for the main comparisons on the primary outcomes. " The authors have conducted some insightful additional analyses which help to demonstrate the robustness of the study findings. | Thanks. | N/A |
| 40 | Overall, the Results are presented accurately and the Discussion is well balanced. | Thanks for this comment. | N/A |
| 41 | "One limitation of this study is that the intervention focused only on red meat, rather than aiming to reduce both red and processed meat. Because Americans have high consumption of processed meat and consumption of processed meat is linked to an array of adverse health effects, future studies should examine how policies impact both red and processed meat. ... At the same time, it is possible that effects of these policies could be attenuated in the real world, where habits, preferences, and other concomitant secular trends (e.g., price inflation) could influence consumer response." Can the authors please expand on the discussion of the study limitations? | Thanks for this comment. We have chosen to omit the discussion about processed meat, since the study was, by design, focused on red meat. A robust discussion on all other categories that might have both health- and environmental-harms (including processed meat as well as other products like sugary drinks or dairy-based desserts) is beyond the scope of the present manuscript. Instead, we have expanded our discussion of the other limitations related to the experimental design and generalizability of the sample, as detailed in our response to comment #28. | Lines 412-432 |
|  | **Reviewer #2** |  |  |
| 42 | Thank you for the opportunity to review this paper which makes a strong and original contribution to the field. How best to proceed with public health interventions on food and drink consumption is a live issue and this paper provides additional robust evidence on the relative benefits of warning labelling and taxation. This is of relevance to policymakers in particular, but also of interest to clinicians experiencing the downstream effects of (lack of) policy. | Thanks for these remarks. | N/A |
| 43 | Data availability - Please ensure that a DOI or relevant identifier is included for the data files prior to publication. | We have now included the DOI for the data files – this link will be made public upon publication (<https://doi.org/10.7910/DVN/HQMFUS>). | N/A |
| 44 | Charts - It may be worth using a different signifier for significant differences as bold text is quite difficult to see, at least at the resolution used in the paper. | We have now updated the chart with asterisks to indicate significant differences. | Tables 1-3  S1-S11 Tables |
| 45 | Line 57 - Please check citation 3. The percentage cited appears to be in the main report but not the appendix. | We have updated citation 3 to be for the main report, not the appendix. | Lines 478-481 |
| 46 | Lines 66-68 - Please consider adding here or in the discussion section a few further references to studies on public acceptability on labelling and taxation (https://doi.org/10.1016/j.socscimed.2019.112395; <https://doi.org/10.1038/s43016-023-00696-y)>; cost-effectiveness of such policies (<https://doi.org/10.1016/j.amepre.2019.02.023>);  simulations of health impact (<https://doi.org/10.3390/nu15041020>); development of economic theory on the subject (https://doi.org/10.1002/ajae.12016; <https://doi.org/10.1086/721078)>; and relevant policy developments (https://www.weforum.org/agenda/2019/08/meat-tax-denmark-sweden-and-germany/; <https://www.fas.usda.gov/data/netherlands-concept-meat-tax-under-discussion-netherlands)>. | Thank you, we have added these references as follows:   - The articles on acceptability and support are referenced in our paragraph on policy implications. - The articles on economic theory are referenced in both the introduction (new theoretical paragraph) and discussion (where we discuss that the impact of taxes was larger than for labels). - The articles on the Germany and Netherlands meat taxes are also referenced in the paragraph on policy implications. - We did not include the article on cost-effectiveness because of its exclusive focus on processed meat.   The paragraph on policy implications reads as follows:  *This study has several public health and policy implications. The results show that taxes and warning labels reduce red meat purchases among red meat consumers, suggesting that adoption and implementation of these policies could yield both health and environmental co-benefits. However, the likelihood of policy adoption is unclear. While several countries in Europe have recently considered adopting meat taxes [18-20], policy discussions on red meat in the US have been limited. A recent legal analysis found that while public policies to encourage meat reduction are legally feasible in the US, political feasibility is highly uncertain given the economic and political power of meat industry lobbies [21]. Policymakers may wish to consider the potential benefits to consumers, along with the legal and political feasibility, when weighing regulatory options for reducing intake of red and processed meat. Another barrier to policy adoption is public support: in this study, overall support for both policies was low (<50% of participants), and similar to* a *previous study [22], support was lower for taxation than labeling. However, some evidence suggests that this barrier could be overcome through different types of messaging about the policy. For example, one study in Germany found that consumers were more likely to support a meat tax if it was justified by animal welfare than by climate change mitigation [23]. Research will be necessary to understand what type of framing or justification is likely to elicit higher support for* m*eat reduction policies in the US.* | Lines 433-448 |
| 47 | Discussion - An additional point that is worth including is the combination of environmental and health warning labels in the study. Future research should separate these out and examine whether either is more effective and whether including more than one has diminishing returns or unintended consequences of creating awareness 'fatigue' (leading to a reduced response). | We have added a discussion of this as follows:  *This study used both health and environmental warnings in the labeling policy condition, so we were unable to disentangle the effects of the two types of warnings. Our previous research has found that health warnings and “combined” warnings that include both health and environmental messages are perceived as more effective than warnings that include only environmental messages [25, 26] suggesting that the health warning may have been responsible for most of the observed reductions in red meat purchases in this study. In contrast, a study in the UK found that nutrition labels (Nutriscore) did not impact the healthfulness of purchases in an online experimental store, but that environmentally-focused “ecolabels”, either alone or in combination with nutrition labels, improved the sustainability of purchases [27]. Future research should examine whether health and environmental warnings have a differential impact on purchasing behaviors, and whether either type of warning is more likely to lead to reactance (message rejection) or label fatigue.* | Lines 401-411 |
|  | **Reviewer #3** |  |  |
| 48 | This manuscript describes an experiment to test the impact of taxes and warning labels on red meat purchases in a virtual (naturalistic) online grocery store. The warning label, 30% tax, and combined warning + tax conditions led to modest reductions in red meat-containing items purchased. The randomized controlled trial design is appropriate and reporting of the study methods and findings was clear and complete. Below are suggestions to increase clarity in places: | Thanks for these remarks. | N/A |
| 49 | Lines 71-72: Secondary objectives included testing the impact of warnings and taxes on psychological measures and support for policies but these were not reported in the abstract. For completeness, I suggest these results are also reported briefly in the abstract. | We have added a brief discussion of the psychological measures to the abstract but could not report the results on policy support due to word limit constraints. | Lines 42-43 |
| 50 | Line 79: Can the authors comment on how representative the 13,000 products in their naturalistic online grocery store were of the full range of products stocked by the grocery retailer they set out to mirror? It would be useful to know if they included products from all departments and aisles or just a selection and, if the latter, how selections were made. | The naturalistic online grocery store contained the full range of foods and beverages stocked by the grocery retailer, excluding alcohol and baby food. The information was obtained by scraping data from a top online food retailer, and all departments and aisles were included. More information about the development of the online grocery store can be found in our previously published protocol [28]. We have added the following text:  *The store included over 13,000 products (****S1 Appendix****), including the full range of foods and beverages stocked by the store and including all departments and aisles.* | Lines 131-132 |
| 51 | Line 85: A purposive sample of 5533 participants was recruited and intended to represent the US population in terms of gender, race/ethnicity, income, and age. However, only 3518 of these (63.6%) completed the study (line 228). The authors should describe how the final sample differed from the intended sample in terms of key demographics and if the final sample was representative of the US population in terms of gender, race/ethnicity, income, and age. Sample demographics are presented in a table but there is no comment on their representativeness. I note that the overall sample was 60% women, 43% college-educated and 73% white so it seems unlikely that it represents the diversity of the US population. this has implications for the generalisability of the study findings to other population groups. | Supplemental Table 2 compares demographic characteristics of randomized participants who completed the shopping task to randomized participants who did not. As can be seen in that table, participants under 60 years of age, who reported doing more than half of their household’s grocery shopping, or who reported doing some but not all of their shopping online were more likely to complete the shopping task. We did not find statistically significant discrepancies between these two groups in terms of gender or red meat consumption.  To provide more insight into generalizability of the sample who completed the shopping task, we have added a supplemental table that compares the study sample to the US adult population overall (**S2 Table**). The analyzed sample has a somewhat smaller share of participants in the 18-29 age group and a somewhat larger share of participants in the 60-69 age group than the general US population, while women were somewhat over-represented in our sample. Also over-represented in our sample were people identifying as white, whereas people identifying as Black or African-American or with another race and people of Hispanic, Latino or Spanish origin were under-represented. As one would expect in similar studies relying on convenience samples, people living in high-income households were also under-represented relative to the US population. We have added acknowledgment of this limitation in the methods and discussion section as follows:  *In the methods:*  *Compared to the US population, our sample had higher representation of women, older adults, people identifying as white and people identifying as not Hispanic, Latino, or Spanish origin (****S2 Table****).*  *In the limitations:* *In addition, our study sample differed in socio-demographic characteristics from the US population (e.g., our sample included somewhat more women and respondents who identified as non-Hispanic white), though it is unclear whether this was due to the online panel, the fact that we included only meat consumers, or both. A related limitation is that, while online food retail is growing, only about 30 to 40% of Americans shop for groceries online [13, 14]. It is not clear whether online food shopping behavior differs from shopping behaviors at traditional retailers, and thus it is unclear how generalizable our results are to consumers who do not shop online. Future research could address these limitations by testing these policies in a real-world brick-and-mortar retail setting where participants use their own shopping lists, spend their own money, and can take home products for consumption.* | Lines 142-144  Lines 424-432 |
| 52 | Line 157: The authors should explain the significance of Cronbach's alpha being greater or less than 0.7 (and hence why outcomes were combined or analyzed separately). | Thank you for this suggestion. We have added the following to the text:  *Reliability of 0.7 is a standard cutoff for demonstrating that items in a scale have sufficient internal consistency [29].* | Lines 208-209  Lines 236-239 |
| 53 | Lines 188-194: What is the clinical significance of a Cohen's f = 0.05 or Cohen's d = 0.13? It would be good to relate these statistics to tangible differences in red meat purchases if possible. | We chose to power for effect sizes that were somewhat smaller than what are typically considered “small” effects (i.e., *f*=0.1 and *d*=0.2). Given the observed mean count and percent of red meat items in the control condition and the observed standard deviations, a Cohen’s *f* of 0.05 corresponds to effects of approximately 0.25 on the count of red meat items (a 7% difference) and 2.7 percentage points on the percent of red meat items. A Cohen’s *d* of 0.13 corresponds to similar effects (0.23 and 2.5, respectively). We have amended the manuscript to reflect this:  *For reference, given the observed mean count and percent of red meat items in the control condition and standard deviations, a Cohen’s f of 0.05 corresponds to effects of approximately 0.25 on the count of red meat items (a 7% difference) and 2.7 percentage points on the percent of red meat items, with similar effects (0.23 and 2.5, respectively) for a Cohen’s d of 0.13* |  |
| 54 | Lines 205-215: Are moderation analyses appropriate when reporting RCT results? The randomized nature of the experiment means personal characteristics should be balanced across groups so I am unsure of the benefit (or indeed appropriateness) of moderation analyses with this study design. They are not usually reported for large public health interventions as far as I am aware. | While the main analysis goal of most RCTs, as you point out, is to estimate an overall effect, there is increasing recognition of the utility of evaluating ‘heterogeneity of treatment effects’ or ‘subgroup analyses.’ Indeed, details of any subgroup analyses are mentioned in the CONSORT checklist for RCTs. A drug trial, for example, might ask: does X drug work as well in women as it does in men? In the context of our study, our goal was to understand whether the impact of the policies on red meat purchases differed for key socio-demographic populations. For example, were the policies more effective among women than men? We have clarified this in the text as follows:  *In order to understand whether taxes and warning labels had a differential effect on red meat purchases for specific socio-demographic subpopulations, we carried out moderation analyses. Specifically, we examined whether intervention effects on the primary outcomes differed by participant characteristics including: red meat consumption in the past 30 days, interest in health, interest in sustainability, household income in the last 12 months, education, age, race/ethnicity, political orientation, and gender (****S2 Appendix****).* | Lines 249-254 |

**References**

1.	Broeks MJ, Biesbroek S, Over EA, Van Gils PF, Toxopeus I, Beukers MH, et al. A social cost-benefit analysis of meat taxation and a fruit and vegetables subsidy for a healthy and sustainable food consumption in the Netherlands. BMC public health. 2020;20(1):1-12.

2.	Springmann M, Mason-D’Croz D, Robinson S, Wiebe K, Godfray HCJ, Rayner M, et al. Health-motivated taxes on red and processed meat: A modelling study on optimal tax levels and associated health impacts. PloS one. 2018;13(11):e0204139.

3.	Säll S, Gren I-M. Effects of an environmental tax on meat and dairy consumption in Sweden. Food Policy. 2015;55:41-53. doi: <https://doi.org/10.1016/j.foodpol.2015.05.008>.

4.	Schönbach J-K, Thiele S, Lhachimi SK. What are the potential preventive population-health effects of a tax on processed meat? A quantitative health impact assessment for Germany. Preventive Medicine. 2019;118:325-31. doi: <https://doi.org/10.1016/j.ypmed.2018.11.011>.

5.	Peñalvo JL, Cudhea F, Micha R, Rehm CD, Afshin A, Whitsel L, et al. The potential impact of food taxes and subsidies on cardiovascular disease and diabetes burden and disparities in the United States. BMC Medicine. 2017;15(1):208. doi: 10.1186/s12916-017-0971-9.

6.	Chalmers N, Revoredo-Giha C, Shackley S. Socioeconomic Effects of Reducing Household Carbon Footprints Through Meat Consumption Taxes. Journal of Food Products Marketing. 2016;22:1-20. doi: 10.1080/10454446.2015.1048024.

7.	Abadie LM, Galarraga I, Milford AB, Gustavsen GW. Using food taxes and subsidies to achieve emission reduction targets in Norway. Journal of Cleaner Production. 2016;134:280-97. doi: <https://doi.org/10.1016/j.jclepro.2015.09.054>.

8.	Koch JA, Bolderdijk JW, van Ittersum K. Can graphic warning labels reduce the consumption of meat? Appetite. 2022;168:105690.

9.	Allcott H, Lockwood BB, Taubinsky D. Should we tax sugar-sweetened beverages? An overview of theory and evidence. Journal of Economic Perspectives. 2019;33(3):202-27.

10.	Taillie LS, Hall MG, Popkin BM, Ng SW, Murukutla N. Experimental Studies of Front-of-Package Nutrient Warning Labels on Sugar-Sweetened Beverages and Ultra-Processed Foods: A Scoping Review. Nutrients. 2020;12(2). Epub 2020/02/27. doi: 10.3390/nu12020569. PubMed PMID: 32098363; PubMed Central PMCID: PMCPMC7071470.

11.	Andrade C. The inconvenient truth about convenience and purposive samples. Indian Journal of Psychological Medicine. 2021;43(1):86-8.

12.	Grummon AH, Tucker AC, Noe V, Rummo PE, Prestemon CE, Hall MG, et al. Consumer behaviour and experiences in a naturalistic online grocery store: implications for nutrition research. Journal of Nutritional Science. 2023;12:e36.

13.	Duffy EW, Lo A, Hall MG, Taillie LS, Ng SW. Prevalence and demographic correlates of online grocery shopping: Results from a nationally representative survey during the COVID-19 pandemic. Public health nutrition. 2022;25(11):3079-85.

14.	Brenan M. In-person grocery shopping rebounds in US; Online also up 2022 [cited 2023 May 26]. Available from: <https://news.gallup.com/poll/397706/person-grocery-shopping-rebounds-online.aspx>.

15.	Frank SM, Taillie LS, Jaacks LM. How Americans eat red and processed meat: an analysis of the contribution of thirteen different food groups. Public Health Nutrition. 2022;25(5):1406-15.

16.	Carl AE, Taillie LS, Grummon AH, Lazard AJ, Higgins IC, Sheldon JM, et al. Awareness of and reactions to the health harms of sugary drinks: An online study of US parents. Appetite. 2021;164:105234.

17.	Grummon AH, Goodman D, Jaacks LM, Taillie LS, Chauvenet CA, Salvia MG, et al. Awareness of and reactions to health and environmental harms of red meat among parents in the United States. Public Health Nutr. 2022;25(4):893-903. Epub 2021/07/30. doi: 10.1017/S1368980021003098. PubMed PMID: 34321133; PubMed Central PMCID: PMCPMC8799779.

18.	van der Hoek M. Concept of a Meat Tax Under Discussion in the Netherlands. United States Department of Agriculture Foreign Agricultural Service, Global Agricultural INformation Network, 2022 Contract No.: NL2022-0037.

19.	Charlton E. This is why Denmark, Sweden and Germany are considering a meat tax: Wold Economic Forum; 2019 [cited 2023 May 26]. Available from: <https://www.weforum.org/agenda/2019/08/meat-tax-denmark-sweden-and-germany>.

20.	Climate change: German MPs want higher meat tax: British Broadcasting Company (BBC); 2019 [cited 2023 May 26]. Available from: <https://www.bbc.com/news/world-europe-49281111>.

21.	Wilde P, Pomeranz JL, Lizewski LJ, Ruan M, Mozaffarian D, Zhang FF. Legal feasibility of US government policies to reduce cancer risk by reducing intake of processed meat. The Milbank Quarterly. 2019;97(2):420-48.

22.	Reynolds JP, Archer S, Pilling M, Kenny M, Hollands GJ, Marteau TM. Public acceptability of nudging and taxing to reduce consumption of alcohol, tobacco, and food: A population-based survey experiment. Social Science & Medicine. 2019;236:112395.

23.	Perino G, Schwickert H. Animal welfare is a stronger determinant of public support for meat taxation than climate change mitigation in Germany. Nature Food. 2023;4(2):160-9.

24.	Hochberg Y, Tamhane AC. Multiple Comparison Procedures. New York: Wiley; 2009.

25.	Taillie LS, Prestemon CE, Hall MG, Grummon AH, Vesely A, Jaacks LM. Developing health and environmental warning messages about red meat: An online experiment. PloS one. 2022;17(6):e0268121.

26.	Taillie LS, Chauvenet C, Grummon AH, Hall MG, Waterlander W, Prestemon CE, et al. Testing front-of-package warnings to discourage red meat consumption: a randomized experiment with US meat consumers. International Journal of Behavioral Nutrition and Physical Activity. 2021;18(1):1-13.

27.	Potter C, Pechey R, Cook B, Bateman P, Stewart C, Frie K, et al. Effects of environmental impact and nutrition labelling on food purchasing: An experimental online supermarket study. Appetite. 2023;180:106312. doi: <https://doi.org/10.1016/j.appet.2022.106312>.

28.	Rummo PE, Higgins I, Chauvenet C, Vesely A, Jaacks LM, Taillie L. A Standardized Guide to Developing an Online Grocery Store for Testing Nutrition-Related Policies and Interventions in an Online Setting. International Journal of Environmental Research and Public Health. 2021;18(9):4527. PubMed PMID: doi:10.3390/ijerph18094527.

29.	Cortina JM. What is coefficient alpha? An examination of theory and applications. Journal of Applied Psychology. 1993;78(1):98-104. doi: 10.1037/0021-9010.78.1.98.
