# Supplementary material for: Impact of taxes and warning labels on red meat purchases among US consumers: A randomized controlled trial
Source: PLoS Med. 2023 Sep 18;20(9):e1004284. doi: 10.1371/journal.pmed.1004284 (PMC10545115; doi:10.1371/journal.pmed.1004284)
Supplement: Attachment — Submitted filename: Response to Reviewers_PLOS Med_Revision 2.docx [file pmed.1004284.s020.docx]

**Ms. Ref. No.:** PMEDICINE-D-23-00922R3

**Title:** “Impact of taxes and warning labels on red meat purchases among US consumers: A randomized controlled trial”

| **#** | **Requests from editors** | **Revision or Response** | **Line Number** |
| --- | --- | --- | --- |
|  | **CT STUDIES** |  |  |
| 1 | Thank you for providing your CONSORT checklist. Please replace the page numbers with paragraph numbers per section (e.g. "Methods, paragraph 1"), since the page numbers of the final published paper may be different from the page numbers in the current manuscript. With my apologies for not having mentioned this in the previous revision round. | We have updated the CONSORT checklist to include paragraph numbers by section. | S1 CONSORT Checklist |
| 2 | In accordance with ICMJE requirements, PLOS Medicine requires prospective, public registration of a data sharing plan (as part of mandatory clinical trials registration) for all clinical trials that began enrollment on or after January 1, 2019. Please confirm the registration of a data sharing plan and add link or add as SI. (Thank you for already adding the Statistical Analysis Plan and Study Protocol). | The data sharing plan is registered on the clinical trials pre-registration. We have updated the text as follows:  *Analyses, hypotheses, and data sharing plan were pre-registered on www.clinicaltrials.gov (NCT04716010) (****S1 Statistical Analysis Plan*** *and* ***S1 Study Protocol****).* | Lines 230-231 |
|  | **TITLE** |  |  |
| 3 | Please add US or similar to your title to provide the locality context of your study. | We have added “US” to the study title. The updated title is as follows:  *Impact of taxes and warning labels on red meat purchases among US consumers: A randomized controlled trial* | Line 1 |
|  | **ABSTRACT** |  |  |
| 4 | For the abstract, please structure the abstracts along the following 3 headings: Background, Methods and Findings, Conclusions. (Currently there are separate headings for Methods and Findings sections). Please remove all other subheaders. | Done. | Lines 28-47 |
| 5 | Please quote aggregate participant demographic characteristics in the abstract; please quote effect size + 95% CI for the primary outcome(s) - not just a p value. For p values, please report p values as p<0.001 and where higher please provide the exact number e.g., as p=0.002 (in line with the format used throughout the manuscript). | We have added aggregate participant demographics and ensured that all primary outcomes included effect size, 95% CI, and a p-value. We have reported p values as p<0.001 or exact when higher.  *Most participants identified as women, consumed red meat two or more times per week, and reported doing all of their household’s grocery shopping.* | Lines 34-43 |
| 6 | Overall, please check and ensure you adhere to the CONSORT abstract checklist (for more details see here: <https://www.equator-network.org/reporting-guidelines/consort-abstracts/)>. | We have reviewed the abstract and confirm that it adheres to the CONSORT abstract checklist. | Lines 25-50 |
| 7 | Last but not least, please add the trial registration number after the abstract. | Done.  *Hypotheses and analyses were pre-registered on* [*www.clinicaltrial.gov*](http://www.clinicaltrial.gov) *(NCT04716010).* | Lines 49-50 |
|  | **ETHICS STATEMENT** |  |  |
| 8 | Please add consent information to the Ethics Statement | We have added the following information to the Ethics Statement.  *All participants provided online written informed consent.* | Line 133 |
|  | **DATA AVAILABILITY** |  |  |
| 9 | Please check if the provided URL is correct: <https://doi.org/10.7910/DVN/HQMFUS> Currently, I receive an error message when trying to access it (DOI cannot be found) although I appreciate you have indicated that this URL will be active upon accept. | The provided URL is correct, and we have now published the link. | N/A |
|  | **AUTHOR’S SUMMARY** |  |  |
| 10 | In the final bullet point of ‘What Do These Findings Mean?’, please include the main limitations of the study in non-technical language. | Done*.*  *The main limitations were that the study differed in socio-demographic characteristics from the US population, and only about 30 to 40% of the US population shops for groceries online.* | Lines 74-75 |
|  | **RESULTS** |  |  |
| 11 | E.g see lines 281 - 282: Suggest reporting statistical information as follows for clarity for the reader “…36%; (95% CI [35%,37%] p=0.001), instead of 36% [(35, 37), p=0.001]. | We have modified the manuscript so that all statistical information is reported as suggested. | Lines 286-289  Lines 303-307  Lines 312-318  Lines 321-325 |
| 12 | Please provide 95% CIs and p values for all results were appropriate, check and amend throughout. | Done. | Lines 286-289  Lines 303-307  Lines 312-318  Lines 321-325 |
| 13 | Please specify the statistical test used to determine p value. | We used fractional probit and Poisson regression models for the primary outcomes and linear regression models for the secondary outcomes. We have added this information throughout the manuscript and supporting information where relevant. | N/A |
|  | **SUPPORTING INFORMATION** |  |  |
| 14 | Can you please re-check figure 3, error bars look identical (which can be correct but maybe have a look if indeed correct). | Thank you for bringing this error to our attention. We have revised the error bars in Figure 3 to show the correct 95% Confidence Intervals. | Figure 3 |
| 15 | I note that S1 Table.doxc still contains branded images - see Survey measures appendix – Sample images.  As before, please confirm you have appropriate permission or you may wish to remove these images. For more information, please see our guidelines: <https://journals.plos.org/plosmedicine/s/figures#loc-licenses-and-copyright> and <https://journals.plos.org/plosmedicine/s/licenses-and-copyright> | We have revised the blurring on the images in S1 Table. If this is not sufficient, we can remove these images from S1 Table. | S1 Table |
|  | **SOCIAL MEDIA** |  |  |
| 16 | To help us extend the reach of your research, if you wish please provide any Twitter handle(s) that would be appropriate to tag, including your own, your coauthors’, your institution, funder, or lab. Please respond to this email with any handles you wish to be included if and when we tweet about this paper. | Please see Twitter handles for co-authors listed below:  @lindseypsmith  @carmenprestemon  @isa_cah_  @AnnaGrummon  @MarissaGHall  @LindsayJaacks  Please see Twitter handle for funder below:  @wellcometrust  Please see Twitter handles for institution and lab:  @GFRP_UNC  @UNCpublichealth  @EdinburghUni | N/A |
